# A novel vector design targeting the blood-brain barrier with the brain-specific AAV-BR1 vector enables abluminal protein secretion from brain endothelial cells in vitro

**DOI:** 10.1186/s12987-025-00738-6

**Published:** 2025-12-12

**Authors:** Bartosz Laczek, Maj Schneider Thomsen, Jakob Körbelin, Annette Burkhart, Torben Moos

**Affiliations:** 1 Neurobiology Research and Drug Delivery, Department of Health Science and Technology, Aalborg University, Selma Lagerlöfs Vej 249, Room 11.02.012, Gistrup, DK-9260 Denmark; 2 ENDomics Lab, Department of Oncology, Hematology, and Bone Marrow Transplantation, University of Medical Center, Martinisstr. 52, 20246 Hamburg, Germany

**Keywords:** Brain gene therapy, Polarized secretion, Abluminal secretion, Blood-brain barrier, Endothelial-specific promoter, C-*Ocln*, Furin cleavage, Viral gene therapy

## Abstract

**Background:**

The blood-brain barrier (BBB) formed by brain endothelial cells (BECs) limits the passage of biopharmaceuticals from the circulation to the brain parenchyma. Genetically modified BECs enable protein secretion to the brain and provide a novel strategy to obtain brain uptake of otherwise BBB-impermeable proteins. However, secretion from BECs may be bidirectional, thereby leading to off-target secretion into the blood. This study investigates a vector strategy using the brain-specific AAV-BR1 viral vector to achieve specific transduction of BECs and to subsequently direct the recombinant proteins towards an abluminal secretion pathway, thereby increasing the release of recombinant proteins into the brain.

**Methods:**

Different AAV-BR1 constructs, under the control of an endothelial-specific promoter (C-*Ocln*), were designed to include a fragment of the platelet-derived growth factor subunit B protein (PDGF-B) fused to the N-terminus of mCherry to promote abluminal secretion. Similar constructs using the more ubiquitous expressed CAG promoter were used for comparisons. The correct processing of the vector constructs was confirmed in vitro, including post-translational removal of the PDGF-B fragment by furin cleavage. AAV-BR1 vector constructs containing the C-*Ocln* promoter and the PDGF-B fragment were systemically administered in C57Bl/6J mice to evaluate abluminal secretion of mCherry to the brain parenchyma specifically by BECs. An in vitro BBB co-culture model based on primary mouse BECs and mixed glial cells was likewise used to evaluate if the PDGF-B fragment enhanced abluminal secretion of mCherry.

**Results:**

The C-*Ocln* promoter exhibited low specificity to BECs, off-target transduction of neurons, and low transduction efficiency, which complicated the in vivo quantification of mCherry secretion into the brain parenchyma. Modelling the BBB in vitro showed higher mCherry secretion across the abluminal membrane when the PDGF-B fragment was included in the vector construct, especially in combination with the CAG promoter.

**Conclusion:**

Investigating abluminal secretion of mCherry was complicated in vivo by high off-target transduction of neurons, despite using the endothelial-specific promoter (C-*Ocln*). In vitro results, however, suggest that incorporating a PDGF-B fragment into the vector design targets the recombinant protein for abluminal secretion by BECs and facilitates increased protein delivery to the brain.

**Supplementary Information:**

The online version contains supplementary material available at 10.1186/s12987-025-00738-6.

## Background

**Fig. 1:**
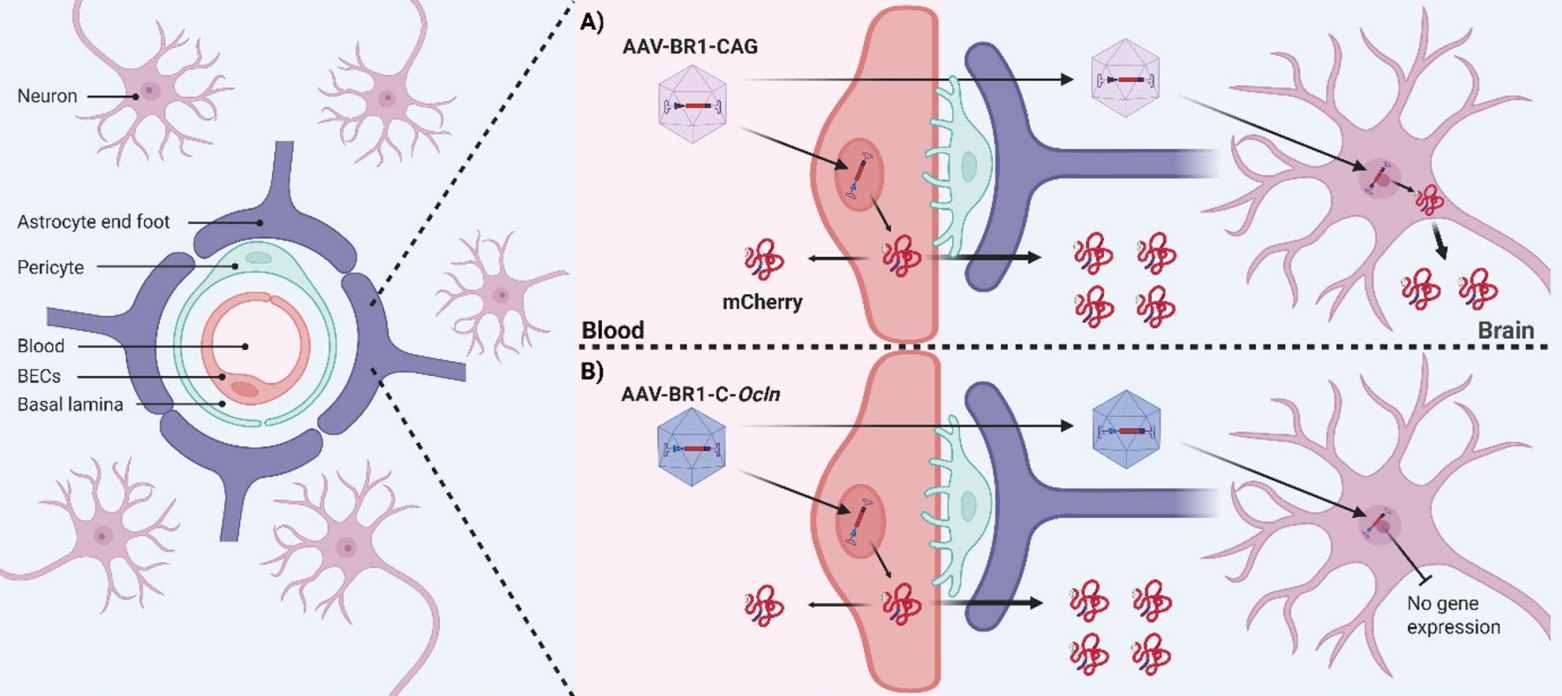
A hypothetical model of targeting only brain endothelial cells to promote abluminal secretion of recombinant proteins. The blood-brain barrier (BBB), located at the level of the brain capillaries, is composed of brain endothelial cells (BECs) lining the vessel wall. BECs are supported by pericytes and astrocytic end feet. **(A)** Genetically modifying the BECs for subsequent gene expression and protein secretion of a recombinant protein, e.g., mCherry, enables delivery of otherwise BBB-impermeable proteins to the brain parenchyma. The adeno-associated viral vector variant (AAV-BR1) specifically transduces BECs but is also able to transverse the BBB and transduce neurons; the latter is mainly seen when the vector construct includes the CAG promoter. Specific transduction of BECs is crucial for studying the secretion of therapeutic proteins into the brain parenchyma, as off-target transduction of neurons results in additional secretion of the therapeutic protein inside the brain parenchyma, thereby obscuring the measurements. **(B)** Replacing the ubiquitous CAG promoter with a synthetic construct consisting of a fragment of the initial enhancer region of cytomegalovirus (CMV) and the occludin promoter, CMV enhancer-occludin (C-*Ocln*), was reported to improve the AAV-BR1 viral vector’s specificity for BECs [13], thereby reducing off-target transduction of neurons. This strategy should enable the measurement of recombinant proteins explicitly secreted into the brain from BECs. Created with BioRender.com

Delivery of therapeutics across the blood-brain barrier (BBB) to the central nervous system (CNS) is a significant bottleneck in the treatment of neurodegenerative disorders [1–3]. Brain endothelial cells (BECs), which make up the BBB, are connected by tight junctions and are closely supported by pericytes and astrocytic end-feet (Fig. 1). One approach to bypass the restrictive nature of the BBB is genetic modification of BECs using viral gene therapy, which enables localized production of biologics and CNS-targeted secretion [4–6]. The AAV-BR1 vector, developed through directed evolution of AAV-2 capsid-modified libraries, is known to primarily target mouse BECs (mBECs), neurons, and pulmonary endothelial cells following intravenous (IV) injection while largely omitting transductions in most peripheral organs, including the liver [5–7].

The strategy of targeting the BBB with gene therapy for treating neurological diseases requires specific transduction of BECs, followed by the production and secretion of therapeutic proteins in the abluminal direction to enable high concentrations of the proteins inside the brain parenchyma. However, monitoring recombinant protein secretion from BECs following gene therapy has encountered several hurdles. Distinguishing between endogenous and recombinant proteins in vivo is technically challenging, as well as determining whether the proteins are produced in response to transduction or have been absorbed by adjacent cells after secretion [5, 6]. Polarized secretion has, therefore, mainly been examined in vitro, and previous attempts primarily resulted in luminal secretion following gene therapy [5, 8]. Another limitation is that some potentially therapeutic peptides, such as glucagon-like peptide 1 (GLP-1), lack a signal peptide for secretion, and N-terminal modifications compromise their bioactivity [9]. Furthermore, the processes driving the polarized secretion within endothelial cells and especially BECs remain poorly understood, with 90% of protein being secreted towards the luminal surface [10–12]. Therefore, the capability of transducing BECs to secrete therapeutic proteins into the brain parenchyma requires further exploration. New vector designs are necessary to circumvent these potential pitfalls.

**Fig. 2:**
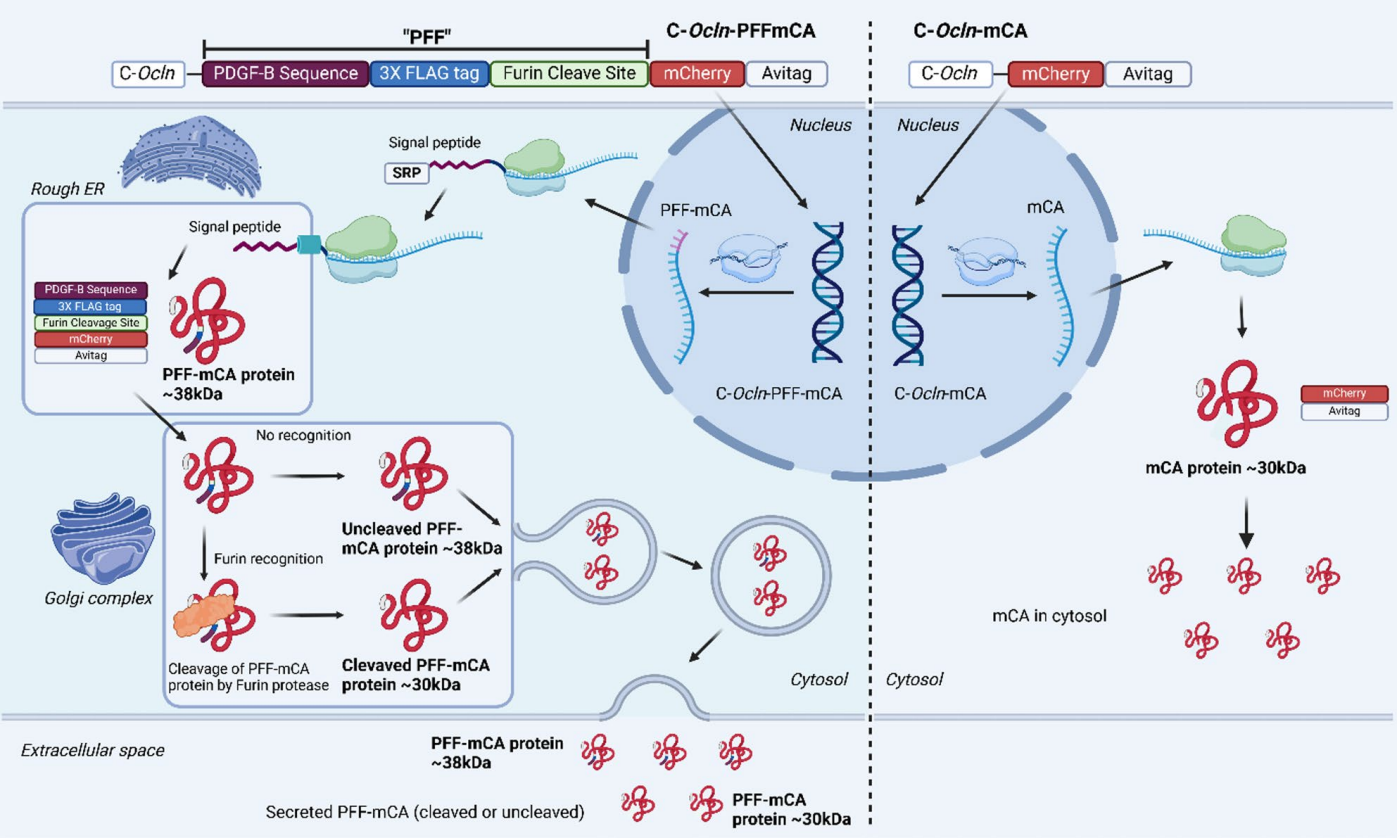
Illustrations of the vector constructs and how they enable secretion of mCherry. The CMV enhancer-occludin (C-*Ocln*) promoter construct is expected to increase the specificity of AAV-BR1 vectors towards brain endothelial cells (BECs). This promoter is therefore included in the vector constructs, C-*Ocln*-PFFmCA and C-*Ocln*-mCA. The difference between the vector constructs lies in the incorporation of a signal peptide from the abluminal secreted protein platelet-derived growth factor subunit B (PDGF-B), including the first 10 amino acids of the protein sequence, to facilitate entry into the secretory pathway directed against the abluminal surface, followed by a 3XFLAG tag and a furin cleavage site recognized by furin protease. Together, these segments are referred to as a PDGF-B-FLAG-Furin (PFF) fragment. Both constructs encode the red fluorescent protein mCherry coupled to an Avitag (mCA). The encoded mCA gene is transcribed into mRNA and translated into recombinant protein. When the PFF sequence is not present, as in the C-*Ocln*-mCA vector (right side), the mRNA is translated into the cytosol, resulting in intracellular accumulation of mCherry-Avitag of an estimated size of ~ 30 kDa. When the N-terminal includes the PFF sequence as in the C-*Ocln*-PFF-mCA vector (left side), mCherry-Avitag is recognized by a signal recognition particle (SRP) and transported to the rough endoplasmic reticulum (ER), where translation is carried out through a translocon incorporated into the rough ER membrane. The protein is folded, the signal sequence of PDGF-B is cleaved, and the protein is transported to the Golgi complex. Here, a furin protease recognizes the furin cleavage site and facilitates cleavage, during which the 3XFLAG tag is lost, resulting in a protein size of ~ 30 kDa, while full-length non-cleaved mCherry-Avitag has a protein size of ~ 38 kDa. Both the cleaved and non-cleaved proteins are transported in vesicles and exocytosed at the cell membrane. Created with BioRender.com

To investigate secretion specifically from BECs in vivo, it is necessary only to transduce BECs, as even moderate transduction of neurons will make it difficult to determine the origin of the protein secretion inside the brain parenchyma (Fig. 1). It was recently shown that substitution of the ubiquitous CAG promoter commonly used in the AAV-BR1 vector constructs [5–7], with a synthetic construct consisting of a fragment of the initial enhancer region of cytomegalovirus (CMV) and the occludin promoter; CMV enhancer-occludin (C-*Ocln*), improved the vector specificity for mBECs and the transduction efficiency in vivo, thereby significantly reducing off-target transduction of neurons and astrocytes [13], as commonly seen using the CAG promoter [5, 6, 13] (Fig. 1). Including a signal sequence that directs the recombinant protein towards an abluminal secretion pathway should increase the protein concentrations in the brain parenchyma, thereby reducing potential off-target effects caused by luminal secretion into the blood. In this study, the protein of interest was, therefore, fused to the signal sequence of the platelet-derived growth factor subunit B (PDGF-B), a protein usually secreted towards the abluminal membrane [14, 15], as this potentially could increase the abluminal secretion of the recombinant protein (Fig. 2).

The present study aims to develop and describe vector strategies for increased abluminal protein secretion in BEC following AAV-BR1-mediated gene therapy. We hypothesized that using the endothelial-specific promoter (C-*Ocln*) and fusion of a non-endogenous fluorescent protein, mCherry, to the PDGF-B fragment would promote the production and subsequent abluminal secretion of mCherry from BECs. Furthermore, we hypothesized that including a furin cleavage site would enable the removal of the PDGF-B fusion sequence after it enters the secretory pathway. We validated and investigated the vector design using immortalized cell lines, describing secretion and furin cleavage. The potential of abluminal secretion from BECs was studied in vivo in mice. Although the endothelial-specific C-*Ocln* promoter was used, tracing mCherry in the brain was complicated by low transduction efficiency and off-target transduction of neurons. The vectors were, therefore, further examined in vitro using BBB models based on primary cells, and their efficiency was compared to similar vector designs incorporating the CAG promoter. The present study demonstrates that incorporating a PDGF-B fragment, combined with a CAG promoter, into the vector design enhances the abluminal secretion of mCherry in vitro.

## Materials and methods

### Strategy for vector design

Two different vector constructs were designed based on the endothelial-specific C-*Ocln* promoter encoding the non-endogenous fluorescent protein mCherry (Fig. 2). The first vector construct was designed for intracellular accumulation of mCherry, and the other vector construct was designed for abluminal secretion of mCherry. mCherry was therefore N-terminally coupled with a fragment of the PDGF-B protein, including its signal sequence, aiming to allow standard processing of the signal peptide and direct the mCherry protein towards an abluminal secretion pathway. Directly downstream of the PDGF-B fragment, a 3XFLAG tag was added for detection purposes, and a furin cleavage site was introduced to analyze the vector’s feasibility for peptides sensitive to N-terminal modifications. The combination of the PDGF-B sequence, 3XFLAG, and furin cleavage is referred to as PFF. mCherry was further integrated with an Avitag (mCA) at the C-terminus to determine if the recombinant proteins had undergone C-terminal or N-terminal truncation. Similar vector constructs were designed with the CAG promoter for comparison. Figure 2 displays the cellular processing of the different vector designs, including potential pathways for the resulting mCherry protein.

### Vector generation and virus production

For the generation of the plasmids C-*Ocln*-PDGF-B-3XFLAG-furin-mCherry-Avitag-WPRE (C-*Ocln*-PFFmCA), and C-*Ocln*-mCherry-Avitag-WPRE (C-*Ocln-*mCA), nucleotide sequences containing the cytomegalovirus (CMV) enhancer-Occludin (C-*Ocln*) promoter were combined with the nucleotide sequences of the PDGF-B signal peptide with its subsequent 10 amino acids, 3XFLAG, furin cleavage site, mCherry, and Avitag (Fig. 2). The plasmid was ordered from GenScript (Piscataway, New Jersey, USA) as complete gene synthesis with insertion into a pFB-1 vector. Vectors containing CAG-PDGF-B-3XFLAG-Furin-mCherry-Avitag-WPRE (CAG-PFFmCA), CAG-mCherry-Avitag-WPRE (CAG-mCA) were obtained by exchanging the C-*Ocln* promoter with the CAG promoter sequence. The Bac-to-Bac expression system was used to package the DNA sequences into the viral AAV-BR1 vector as previously described [5].

### Cell culture

HEK-293T and bEnd.3 cells (purchased from ATCC, maximum passage number #P15) were cultured in T25 flasks with complete Dulbecco’s Modified Eagle Medium (cDMEM) containing DMEM, high glucose (Thermo Fischer Scientific, cat#11965092) supplemented with 10% Fetal Calf Serum (FCS) (Life Technology, cat#10270), 2mM L-glutamine (Thermo Fischer Scientific, cat#25030081) and 5 mg/L gentamicin sulfate, (Thermo Fischer Scientific, cat#15750060). For experiments, cells were seeded in 24-well plates.

As previously described, MBECs and glial cells were isolated for in vitro BBB modeling [16, 17]. Before co-culturing with mBECs, glial cells were seeded into 12-well plates and cultured without passaging for two weeks, with medium change every four days to obtain confluent monolayers. mBECs were cultivated in mBECs-medium, containing DMEM-F12 (Life Technology, cat#31331) supplemented with 10% plasma‐derived bovine serum (First Link, cat#60‐00‐810), 10 µg/ml Insulin transferrin sodium selenite (Merck KGaA, cat#11074547001), 10 µg/ml gentamicin sulfate, 1 ng/µl freshly added basic fibroblast growth factor (PeproTech Nordic, cat#100‐18B). For co-cultures, mBECs were seeded at an estimated density of 100,000 cells/cm2 onto 12-well hanging filter inserts with 1 μm pore membranes (Greiner Bio-One, cat#665610) pre-coated twice with collagen IV and fibronectin at a final concentration of 250 µg/ml and 100 µg/ml, respectively. The subsequent day, the hanging filter inserts with mBECs were transferred to 12‐well plates containing glial cells. Tight junction formation was induced by exchanging medium in the top chamber with mBECs-medium supplemented with 250 µM CTP‐cAMP (Merck KGaA, #C3912), 17.5 µM RO (Merck KGaA, #B8279), and 550 nM hydrocortisone (Merck KGaA, #H4001), and in the lower chamber with medium containing a 1:1 ratio of glial conditioned medium and mBECs-medium supplemented with 550 nM hydrocortisone. Daily measurements of barrier integrity were conducted using trans-endothelial electrical resistance (TEER) measurements with a MiliCell ERS-2 epithelial volt-ohm meter and an STX01 chopstick electrode (Millipore, cat#MERSSTX01). The final TEER values were calculated as Ω × cm^2^ by subtracting TEER readings from cell‐free double-coated hanging filter inserts from co-culture TEER readings and multiplying the outcome with the filter area (1.12 cm^2^).

### Transfection of immortalized cell lines

The human HEK 293T and mouse bEnd.3 cell lines were seeded in 24-well plates and allowed to reach approximately 60–70% confluency in cDMEM. Both cell lines were free from mycoplasma contamination but had not recently been authenticated. Two hours before transfection, the medium was changed. Polyethylenimine, Linear, MW 25,000, Transfection Grade (PEI 25 K™) (Polysciences, cat#23966-100) was used as transfection reagent in a 3:1 weight ratio of plasmid DNA used in a concentration of 1 µgDNA/one million cells. PEI and plasmid DNA were separately mixed with Opti-MEM (Thermo Fischer Scientific, cat#31985062) before the PEI mixture was added to the DNA mixture, thoroughly vortexed, and incubated at room temperature (RT) for 20 min. After incubation, the PEI/DNA complex in Opti-MEM was added to the cells by carefully pipetting in droplets and carefully mixing the plate in a circular motion. The medium was harvested 72 h post-transfection and centrifuged for 5 min at 2000 x g. The supernatant was collected into a new tube. For statistical analysis, cells originating from three distinct frozen cell aliquots were transfected on three separate occasions, using two technical replicates per experiment, resulting in an n-value of 6.

### Transduction of primary cells

mBECs were transduced by adding 105 vg/cell of either AAV-BR1-C-*Ocln*-mCA, AAV-BR1-C-*Ocln*-PFFmCA, AAV-BR1-CAG-mCA, or AAV-BR1-CAG-PFFmCA to the hanging filter insert containing mBECs, one day after co-culturing with glial cells. The addition of the virus to the upper chamber represents a model of a systemically administered virus with mBECs on hanging filter inserts representing the BBB and the mixed glial cells representing the brain parenchyma. Transduction of mBECs in co-culture with glial cells was repeated three times at different time points using primary cells derived from different aliquots and isolations. Four days post-transduction, medium from the upper and lower chambers was harvested and centrifuged for 5 min at 1000 x g, and the supernatant was transferred to a new tube. The medium was frozen for later ELISA analysis. mBECs and glial cells were washed twice with PBS, fixed with 4% paraformaldehyde for 5 min, and washed twice in PBS. Fixated cells were stored in PBS at 4 °C for later immunocytochemical analysis.

### Animals for in vivo experiments

36 eight-week-old female C57Bl/6J mice were purchased from Janvier Lab. Upon arrival at the animal facility, the mice were acclimatized for a minimum of 14 days under local environmental conditions, which included a temperature range of 20–24 °C, relative humidity between 40% and 60%, and a 12-hour light/dark cycle. The mice were housed in standard cages with water, and a commercial diet (Altromin 1324, Brogaarden, Gentofte) provided ad libitum. Mice were monitored for weight changes every second week and daily for well-being. Weight loss exceeding 20% or indications of deteriorating health were defined as humane endpoints resulting in the humane euthanizing of the mice. No mice were excluded from the experiment before the final termination date. Animal experiments were conducted in compliance with ethical regulations at Aalborg University following the Danish experimental permit 2018-15-0201-01467. The animal study is reported according to the ARRIVE guidelines [18].

### Experimental design for in vivo transduction and sample extraction

The mice were randomly assigned to three groups without blinding and IV injected through the tail vein with either PBS (*n* = 8), AAV-BR1-C-*Ocln*-mCA (*n* = 14), or AAV-BR1-C-*Ocln*-PFFmCA (*n* = 14). Each mouse was administered a dose of 1∙10^11^ vg in a total volume of 100 µl. Animals were monitored for 30 min. post-injection for signs of hypersensitivity. Transgene expression following AAV-BR1 transduction peaks after 20–40 days, although high levels of expression are sustained for years [7]. For this study, mice were sacrificed on day 28, as we were interested in examining the animals when the high quantities of recombinant proteins were obtainable. On day 28, the mice were anesthetized via subcutaneous injections of Hypnorm/Dormicum (0.063 mg fentanyl; 2 mg fluanison; 0.1 mg Dormicum). Cerebrospinal fluid (CSF) was collected by exposing the cisterna magna through an incision and inserting a glass capillary to extract the CSF (PBS (*n* = 4), AAV-BR1-C-*Ocln*-mCA (*n* = 6), and AAV-BR1-C-*Ocln*-PFFmCA (*n* = 6)). Blood samples were collected intracardially from all mice using a heparin-flushed syringe, followed immediately by transcardiac perfusion with 20 mL of potassium PBS (PPBS). The collected blood samples were kept on ice until centrifugation for 10 min at 2000 × g at 4 °C, to separate the plasma. Two mice from the PBS control and three from each of the AVV-BR1 experimental groups underwent transcardiac perfusion with 20 ml of 10% formaldehyde after perfusion with PPBS. Subsequently, brains were extracted and stored in 10% formaldehyde overnight. The fixated brain tissue was washed in PPBS followed by submersion in a 30% ucrose solution for three days before sectioning into 40 μm coronal sections at -21 °C in a cryostat (Leica CM3050 S, Germany), after embedding with Tissue-Tek^®^ O.C.T.™ Compound (Sakura, cat# 4583). Brain sections were distributed into six equivalent sets and stored in an anti-freeze solution at -20 °C. The remaining brains not perfused with formaldehyde (PBS (*n* = 6), AAV-BR1-C-*Ocln*-mCA (*n* = 11), and AAV-BR1-C-*Ocln*-PFFmCA (*n* = 11)) were divided into a left hemisphere used for capillary depletion and a right hemisphere, which were snap-frozen on dry ice for further biochemical analysis.

### Capillary depletion

The left hemispheres were placed in a Dounce homogenizer with 525 µl ice-cold capillary depletion buffer (0.01 M HEPES, 0.14 M NaCl, 0.004 M KCL, 0.0028 M CaCl, 0.001 M MgSO_4_, 0.001 M NaH_2_PO_4_, 0.01 M D-glucose), and homogenized by six strokes followed by the addition of 1000 µl of ice-cold 30% dextran solution and six additional strokes. A 200 µl aliquot of the total brain homogenate was immediately snap-frozen for RT-qPCR analysis, while the remaining homogenate was kept on ice and centrifuged at 4 °C for 40 min at 5400 x g with acceleration and deceleration curves adjusted to 9 and 5, respectively, to separate the capillary-enriched tissue from the capillary-depleted brain tissue. The capillary-enriched and depleted brain tissues were separated by scooping the top layer (capillary-depleted tissue), the in-between layer was discarded by carefully pipetting, and the pellet containing capillary-enriched tissue was without further processing (including washing steps) stored at -70 °C for further biochemical analysis.

### Western blotting

Medium from bEnd.3 and HEK 293T cells transiently transfected with PEI and plasmids of interest were subjected to western blotting. The medium was diluted with 4X LDS Sample buffer (Invitrogen, #cat NP007) and 10X Sample Reducing Agent (Invitrogen, #cat NP005), followed by a 5-minute denaturation at 95 °C. Samples and the Chameleon Duo Prestained Protein Ladder (Li-Cor, cat# 928–6000) were added to a 4–12% Bis-Tris gel (Invitrogen, #cat NP0323) and subjected to 120 V for 90 min. Protein transfer from the gel onto a nitrocellulose membrane (Invitrogen, #cat LC2001) using NuPAGE transfer buffer (Invitrogen, #cat NP0006), at 6 V for 16 h. The membranes were blocked in a 5% skimmed milk solution in tris-buffered saline with Tween^®^ 20 (TBS-T) for 1 h, followed by the addition of the primary antibodies, rabbit anti-Avitag 1:1000 (Genscript, cat# A00674) or rabbit anti-FLAG 1:1000 (Merck KGaA, cat#F7425-2MG) in a 1% skimmed milk solution in TBS-T at 4 °C overnight on a rocking table. The membranes were washed three times with TBS-T before adding an HRP-conjugated anti-rabbit secondary antibody (1:2000) (Santa Cruz, cat# sc-2004) in 1% skimmed milk in TBS-T for 1 h at RT. After three consecutive washes with TBS-T, the membranes were subjected to Pierce ECL western blotting substrate (Thermo Fisher Scientific, cat#32109) for 5 min and imaged using an LI-COR Odyssey FC system. Membranes containing medium samples from cells transfected with plasmids containing a CAG and C-*Ocln* promoter were measured for chemiluminescence for 3 min and 50 min, respectively. Uncropped western blots can be seen in Supplementary Figures S1 and S2.

### Enzyme-linked immunosorbent assay

All ELISA measurements in this study were conducted using an ELISA KIT specific to mCherry (Abcam, cat# ab221829) following the manufacturer’s protocol. In short, all samples were analyzed in duplicates, and cell culture medium and extraction reagents without cell contact were also measured to standardize sample absorbance. The standards containing mCherry ranged from 6.25 to 400 pg/ml, and absorbance was measured at 450 nm. Duplicate measurements varying above 0.4 in absorbance were excluded from further analysis. Concentration in the samples was determined by normalizing the absorbance to the corresponding medium and then applying the linear regression from the standardized samples. To remain within the quantitative range of the kit, the medium from transiently transfected cultures was diluted 1:50 and 1:5 for medium from transfections performed with a plasmid containing the CAG and C-*Ocln* promoters, respectively. The medium from the co-culture transduced with AAV-BR1-CAG-PFFmCA was diluted 1:10 to remain within the quantitative range. For measurements of mCherry content in capillary-enriched (capillary) and capillary-depleted (parenchyma) fractions, the samples were lysed according to the manufacturer’s protocol using Neuronal Protein Extraction Reagent (N-PER) (Thermo Fisher Scientific, cat# 87792). For standardization of ELISA measurements from the lysed samples, total protein concentration was determined using the Pierce BCA Protein Assay Kit (Thermo Fisher Scientific, cat# 23225) according to the manufacturer’s protocol. Raw data from the ELISA analysis are included in Supplemental Figure S3.

### Immunohistochemistry

Free-floating cryosectioned brain slices were incubated in a blocking buffer containing 3% porcine serum and 0.3% Triton-X100, diluted in potassium phosphate-buffered saline (PPBS) for one hour at RT. Subsequently, the brain slices were incubated with the primary antibodies rabbit anti-mCherry (1:500, Rockland, cat# 600-401-P16), in combination with either rat anti-cluster of differentiation 11b (CD11b) (1:200, Bio-Rad, cat#MCA711G), mouse anti-NeuN (1:500, Chemicon, cat# MAB377), or rat anti-mouse transferrin receptor (TfR) (1:100, Fischer Scientific, cat# 15227067), diluted in blocking solution and left overnight at 4 °C with gentle agitation. The slices were washed three times in a washing buffer (blocking buffer diluted 1:50 in PPBS), before incubation at RT for one hour with gentle agitation with the secondary antibodies anti-mouse Alexa Fluor 488 (1:200, Invitrogen, cat# A21202), anti-rat Alexa Fluor 488 (1:200, Invitrogen, cat# A11006) and/or biotinylated goat anti-rabbit (1:200, Vector, cat# BA-1000) diluted in blocking buffer. Sections stained with a biotinylated conjugate were incubated with a solution containing two drops of reagent A and B of a Vectastain kit in 10 ml PPBS (Vector, cat# PK-6103) for 30 min and washed three times in PPBS before incubating with Tyramide (1:50, Perkin Elmer, cat# NEL 700A001KT), followed by three washes in PPBS and another round of Vectastain incubation for 30 min and three washes in PPBS. These sections were then incubated with streptavidin Alexa Fluor 568 (1:200, Thermo Fisher Scientific, cat# S11226) at RT for one hour with gentle agitation. Then all sections were washed twice in washing buffer and twice in PPBS. Finally, the slices were incubated with 2 µg/mL 4′,6-diamino‐2‐phenylindole (DAPI) in PPBS for 5 min, followed by a final wash in PPBS. Sections were mounted on microscope glass slides with a DAKO fluorescent mounting medium (DAKO, #S3023). Bioimaging was conducted on an Olympus IX83 inverted microscope with a Yokogawa CSU-W1 spinning disk unit or a confocal laser scanning microscope ZEISS LSM900 (Carl Zeiss).

### Immunocytochemistry

Primary mBECs and mixed glial cells were incubated with blocking buffer containing 5% milk, 0.3% Triton-X100 (Merck KGaA, cat# X100), and diluted in PBS for 1 h at RT. Next, primary antibodies diluted in blocking buffer were added and incubated at 4 °C overnight with gentle rocking. mBECs were labeled with rabbit anti-Avitag 1:300 (Genscript, cat# A00674) and mouse anti-ZO-1 1:200 (Invitrogen, cat# 339100), while mixed glial cells were labeled with rabbit anti-Avitag 1:300 and rat anti-CD11b 1:200 (Bio-Rad, cat# MCA711G). Subsequently, the cells were washed three times in PBS, and secondary antibodies diluted 1:500 in blocking buffer were added for 1 h at room temperature with gentle rocking. The secondary antibodies included anti-rabbit Alexa Fluor 568 (Invitrogen, Cat# A11011), anti-rat Alexa Fluor 488 (Invitrogen, cat# A11006), and anti-mouse Alexa Fluor 488 (Invitrogen, cat# A21202). Finally, cell nuclei were stained with 2 µg/ml DAPI in PBS and mounted on microscopy slides with DAKO fluorescent mounting medium. Bioimaging was conducted on a confocal laser scanning microscope ZEISS LSM900 (Carl Zeiss).

### Bioimage analysis

All image processing was conducted in FIJI [19]. Image contrast was adjusted to minimize unspecific noise. Z-stacks were all compiled by maximum intensity projections.

### Reverse transcription-quantitative polymerase chain reaction

RNA was extracted from 30 mg brain homogenate from each mouse using the GeneJET RNA purification kit (Thermo Fisher Scientific, cat# K0732) following the manufacturer’s protocol for extraction from mammalian tissue. Following RNA extraction, samples were treated with DNase I (Thermo Fisher Scientific, cat# EN0525) for 30 min at 37°C. EDTA was added to the reaction and terminated at 65°C for 10 minutes. Following the manufacturer’s instructions, the DNase-treated RNA samples were used as templates for cDNA synthesis using the Maxima H Minus First-strand cDNA Synthesis Kit (Thermo Fisher Scientific, cat# K1651). The qPCR reaction was conducted using the FastStart Essential DNA Green Master (Roche, cat#06402712001), based on SYBR green, and primers specific towards mCherry (Fw; 5’-CCA AGC TGA AGG TGA CCA AG-3’, Rev; 5’-GTC CTC GAA GTT CAT CAC GC-3’), actin beta (*Actb*) (Fw; 5’-CTG TCG AGT CGC GTC CAC C-3’, Rev; 5’-TCG TCA TCC ATG GCG AAC TGG-3’), and hypoxanthine phosphoribosyltransferase 1 (*Hprt1*) (Fw; 5’- GTT GGA TAC AGG CCA GAC TTT GTT G’, Rev; 5’- GAT TCA ACT TGC GCT CAT CTT AGG C-3’). Reactions were performed with 2 ng cDNA and 3 µM primers. Primer efficiency was evaluated based on a tenfold serial dilution of samples using the equation E = 10(-1/slope). The thermal profile of the analysis was 10 min at 95 °C, followed by 40 cycles of 95 °C for 15 s and 60 °C for 1 min. The melt curve thermal profile consisted of 95 °C for 15 s, descending to 60 °C for 1 min, and ascending back to 95 °C. All measurements were conducted using Quant Studio 6 Flex (Applied Biosystems, cat# 4485699). Samples were analyzed in triplicate for each primer, and non-reverse-transcribed RNA and no-template controls served as negative controls. If a sample had an incoherent triplicate measurement (>0.5 in threshold cycle), a signal in the negative controls, or abnormal melt curve peaks, it was excluded from further analysis. The geometric mean of the reference genes *Actb* and *Hrpt1* was used for normalization, and the relative mRNA expression was calculated using the Pfaffl method [20].

### Statistics

All statistics in this study, except power analysis, were conducted using GraphPad Prism 9.5.1 (GraphPad Software, Boston, Massachusetts, USA, www.graphpad.com). The sample size was calculated using IBM SPSS Statistics (Version 28) with a power analysis based on data from three mice, suggesting a minimum of six mice per group to achieve 80% power in the statistical analysis of protein quantities in capillary and brain tissue. No animals were excluded from the study. All datasets were analyzed for normality using a Shapiro-Wilk test and a QQ plot. Groups with normally distributed data were investigated with an F-test to compare variances. When the F-test was passed, the data sets were analyzed using the appropriate test for parametric data and presented as mean ± standard deviation values. Non-parametric data were presented with a median and 95% confidence interval (CI). Statistical analysis for a given dataset is described in the figure legends. For in vivo experiments, n-values represent the total number of mice included in the study. For in vitro experiments, n-values equate to one well, and each experiment was repeated three times. Significance is set at p-values of *P ≤ 0.05, **P ≤ 0.01, ***P ≤ 0.001, and ****P ≤ 0.0001.

## Results

To investigate the capability of BECs to secrete recombinant proteins into the brain parenchyma, two plasmid constructs were designed to encode the fluorescent protein mCherry with and without the initial segment of the PDGF-B sequence, theoretically allowing for abluminal protein secretion under the control of the endothelial-specific C-*Ocln* promoter. In addition, a furin cleavage site was included to assess the efficiency of N-terminal sequence cleavage. Evaluating the furin cleavage is used as an indirect measure of the recombinant protein being sorted into the secretory pathway, as furin cleavage typically occurs in the Golgi apparatus. The 3XFLAG tag is included upstream of the furin cleavage site and should therefore only be present in uncleaved mCherry protein (Fig. 2). The combination of the PDGF-B sequence, 3XFLAG, and furin cleavage is referred to as PFF. For a better comparison of the results to previous studies [4–7, 13], similar plasmid constructs were designed by replacing the endothelial-specific C-*Ocln* promoter with the ubiquitous CAG promoter. The expected mechanisms and locations of signal sequence recognition, furin cleavage, and secretion, with resulting protein sizes of 38 kDa for the non-cleaved mCherry and 30 kDa for the cleaved mCherry, are explained in Fig. 2.

### The presence of the PFF sequence leads to furin processing and secretion of mCherry

**Fig. 3:**
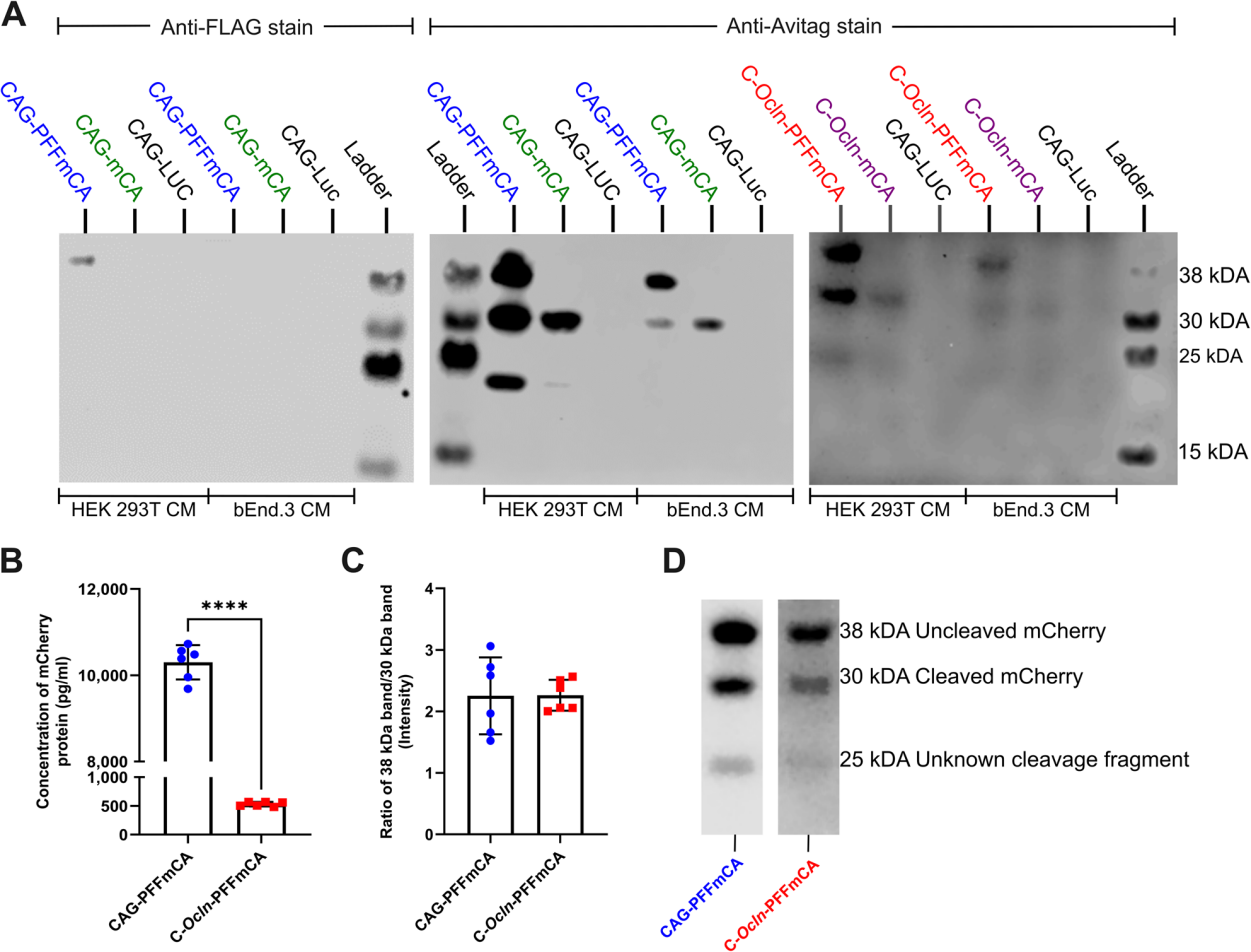
mCherry-Avitag detection in medium from transfected HEK 293T and bEnd.3 cells. **(A)** Western blots were conducted on cell culture medium (CM) collected from HEK 293T or bEnd.3 cells post-transient transfection with vector constructs encoding mCherry-Avitag (mCA) with and without the PDGF-B-3XFLAG-Furin (PFF) fragment under the control of CAG or CMV enhancer-occludin (C-*Ocln*) promoter. A plasmid encoding Luciferase under the control of the CAG promoter (CAG-Luc) was included as a negative control. The ladder represents 38, 30, 25, and 15 kDa bands. Anti-FLAG staining of HEK 293T cells transfected with CAG-PFF-mCA reveals the full-length construct at 38 kDa, which is also detected using an anti-Avitag antibody. mCA is additionally recognized at ~ 30 kDa, and just below 25 kDa. The 30 kDa protein band corresponds to the cleaved mCA product recognized by furin proteases, while the 25 kDa band might be an unknown N-terminally truncated fragment of the mCherry-Avitag. The exposure time on the left membrane is 3 min, while the exposure time on the right membrane is 50 min, indicating much higher concentrations of mCherry secreted by HEK cells. Uncropped western blots can be seen in Figure S1. **(B)** The concentration of mCA secreted into the medium is significantly higher in bEnd.3 cells transfected with CAG-PFFmCA compared to bEnd.3 cells transfected with C-*Ocln*-PFFmCA. Data are presented as the mean ± SD (*n* = 6). A student t-test was used for statistical analysis (****P < 0.0001). **(C)** The ratio of cleaved products was determined by measuring the relative intensity of the 38- and 30-kDa bands in anti-Avitag western blots performed on culture medium from transfected bEnd.3 cells. Data is presented as mean ± SD (*n* = 6). Student t-test was used for statistical analysis (*P* = 0.9741). **(D)** A representative western blot image obtained using medium from bEnd.3 cells following transient transfection with CAG-PFFmCA and C-*Ocln*-PFFmCA vector constructs. Uncropped western blots can be seen in Figure S2

HEK 293T and bEnd.3 cells were transiently transfected with the four different constructs to confirm their function and to evaluate whether the PFF sequence directs mCherry towards a secretory pathway, as well as its sensitivity to furin cleavage. Medium from the transfected cells was subsequently collected and analyzed by western blotting to detect secreted mCherry. Transfected HEK 293T cells produced much higher quantities of mCherry than bEnd.3 cells, making it difficult to simultaneously visualize the secreted proteins in the medium from both cell types (Fig. 3A). Including the PFF sequence in the vector constructs results in a distinct cellular processing of mCherry in both cell types. An anti-FLAG-tag-staining of medium from HEK 293T reveals a 38 kDa band corresponding to the complete protein product of PFFmCA, which has not been subjected to furin cleavage. Anti-Avitag-staining revealed three bands, at approximately 38, 30, and below 25 kDa, in the medium from both HEK293T and bEnd.3 cells transfected with the CAG-PFFmCA or C-*Ocln*-PFFmCA constructs (Fig. 3A). These correspond to the complete full-length non-cleaved mCherry protein (38 kDa), and the furin-cleaved mCherry, where the 3XFLAG tag is removed (30 kDa), showing that both cell types can recognize the furin cleavage site. No positive anti-FLAG-tag staining was observed in the bEnd.3 cells, despite the anti-Avitag staining, indicate that these cells also produce a full-length mCherry protein. This is probably due to the lower transfection efficiency of bEnd.3 compared to HEK 293T cells, which makes the protein quantity secreted from bEnd.3 too low for detection of the 3XFLAG tag. This is also supported by the weak anti-Avitag staining, where the quantity of full-length mCherry protein is higher in HEK 293T cells transfected with CAG-PFFmCA compared to bEnd.3 cells transfected with the same vector. The band below 25 kDa is suggested to be an unknown N-terminally truncated cleavage fragment of mCherry, due to the retained C-terminal Avitag (Fig. 3A). When the cells were transfected with vectors not containing the PFF sequence, it was expected that mCherry would be contained intracellularly and therefore not be detected in the medium (Fig. 2). However, a 30 kDa band was detected in medium from cells transfected with both non-secretory vector constructs (CAG-mCA or C-*Ocln*-mCA), however in a lower quantity than observed using similar vector constructs containing the PFF sequence, suggesting unspecific release of intracellular proteins into the medium. A significant number of cells die following the transfection procedure, so detecting some amount of mCherry in the medium due to cell lysis is expected, even though the vector construct is not designed to enable secretion. It could also be due to a non-specific secretory mechanism, possibly resulting from high intracellular concentrations of mCherry, especially in HEK 293T cells. Transfection with CAG-Luc (encoding Luciferase) was included as a negative control for the anti-FLAG tag and anti-Avitag staining. The CAG promoter appears to be superior to the C-*Ocln* promoter, consistent with a previous in vitro study [13]. However, these results were not replicated when the same promoters were compared in vivo, where the C-*Ocln* promoter was identified as the better choice [13]. The validation of the vector constructs was performed using transfection, as the AAV-BR1 vector was found to be ineffective in effectively transducing bEnd.3 cells.

### Furin cleavage efficiency is not affected by the quantity of protein secretion in bEnd.3 cells

HEK 293T cells appear to be more effective in producing furin-cleaved products than bEnd.3 cells, as indicated by the ratios between the full-length 38 kDa PFFmCA product and the furin-cleaved 30 kDa PFFmCA products (Fig. 2A). With furin cleavage being possibly limited in BECs, we wanted to investigate if the ratio of cleavage was affected by the quantity of secretion. Recognizing the high expression in vitro using the CAG promoter, we attempted to determine if the higher secretion would result in a lower cleavage ratio. bEnd.3 cells were therefore transiently transfected with either CAG-PFFmCA or C-*Ocln*-PFFmCA. The medium was collected, and total mCherry protein concentration was measured using ELISA (Fig. 3B), while the ratio of mCherry cleavage products was analyzed using an anti-Avitag western Blot (Fig. 3C, D). Medium collected from bEnd.3 transfected with CAG-PFFmCA resulted in significantly higher mCherry concentrations (10,301 ± 397 pg/ml) than the medium collected from bEnd.3 transfected with C-*Ocln*-PFFmCA (531 ± 39 pg/ml) (Fig. 3B). When analyzing the ratios of the full-length and the furin-cleaved mCherry-Avitag protein products, there was no statistical difference identified between the two promoter types (Fig. 3C), indicating that the likelihood of furin cleavage in BECs is not affected by the quantity of the protein being secreted.

In summary, the validation of the constructs demonstrates that the PFF sequence yields higher quantities of mCherry secreted into the cell culture medium and that the CAG promoter results in higher in vitro expression of mCherry compared to the C-*Ocln* promoter. Furin cleavage does not seem to be very efficient in bEnd.3 cells, but the efficiency is not affected by the quantity of secreted mCherry protein.

### Transduction of mice with AAV-BR1-C-*Ocln*-mCA and AAV-BR1-C-*Ocln*-PFFmCA leads to mCherry expression in both BECs and neurons

To specifically investigate abluminal protein secretion from mBECs in vivo, the brain-specific AAV-BR1 viral vector was incorporated with the C-*Ocln-*mCA *and* C-*Ocln-*PFFmCA constructs. The C-*Ocln* promoter was chosen for in vivo investigations due to its higher mBECs specificity [13], which was expected to allow for differentiation between abluminal mCherry secretion originating from mBECs and mCherry expressed locally within the brain parenchyma, e.g., via off-target transduction of neurons (Fig. 1). High concentrations of mCherry within the brain parenchyma would be expected when using the ubiquitous CAG promoter, as this promoter also allows for strong gene transcription in transduced neurons and astrocytes [5, 6, 13], potentially obscuring the identification of mCherry derived from BEC by abluminal secretion (Fig. 1). Mice were therefore injected with either AAV-BR1-C-*Ocln*-mCA, AAV-BR1-C-*Ocln*-PFFmCA, or PBS (CTRL) 28 days later, the brain, plasma, and CSF were collected.

**Fig. 4:**
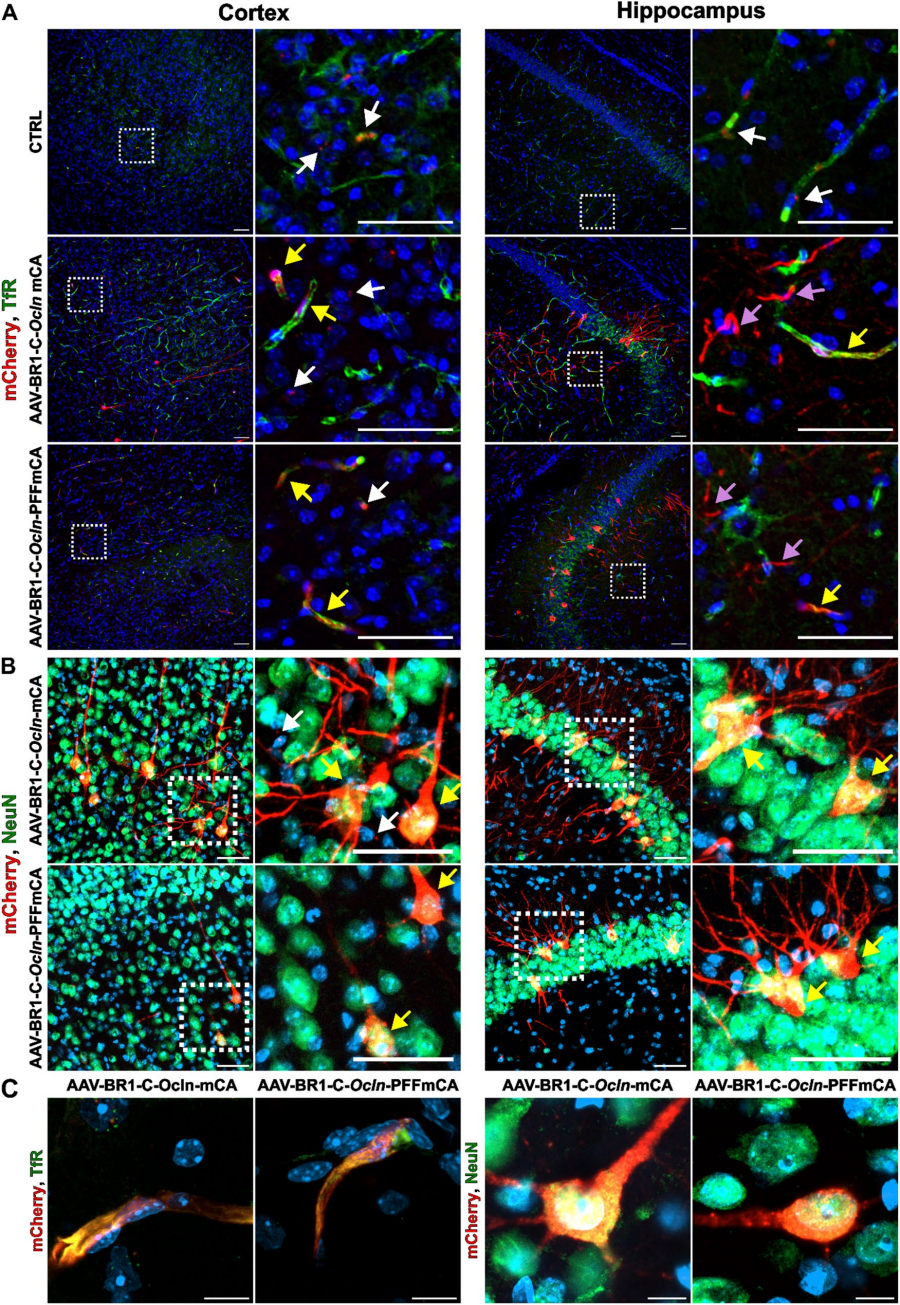
mCherry co-localizes both brain endothelial cells (BECs) and neurons. **(A)** Transduced cells were immunohistochemically stained for mCherry (red), and co-stained for transferrin receptor (TfR) (green) to identify BECs in the cerebral cortex (left) and hippocampus (right). mCherry colocalizes with TfR, indicating successful transduction of BECs (yellow arrows); however, some transduced cells failed to co-localize with mBECs (TfR) (magenta arrows). Areas marked with dashed lines are shown in higher magnification. Patterns of nonspecific fluorescence, interpreted as fluorescence-emitting lipofuscin granules, appear as small circular red dots and are easily identified in all groups, particularly in control (CTRL) mice (white arrows). No distinct differences are observed between mice transduced with AAV-BR1-C-*Ocln*-mCA or AAV-BR1-C-*Ocln*-PFFmCA. **(B)** mCherry also colocalizes with the neuronal marker, neuronal nuclear antigen (NeuN) (green), indicating off-target transduction of neurons (yellow arrows) in both the cerebral cortex and hippocampus. mCherry colocalizes with neurons in mice transduced with both AAV-BR1-C-*Ocln*-mCA and AAV-BR1-C-*Ocln*-PFFmCA. The nuclei are counterstained with DAPI (blue). Scale bar = 50 μm. **(C)** Images of a single transduced BEC (left side) and a neuron (right side) at high magnification. No difference is observed in the intracellular localization of mCherry in the cells. Scale bar = 10 μm. All images are representative of mice transduced with either AAV-BR1-C-*Ocln*-mCA (*n* = 3), AAV-BR1-C-*Ocln*-PFFmCA (*n* = 3), or CTRL mice (*n* = 2)

To investigate the presence and potential differences in the localization of mCherry within the brain of mice transduced with the AAV-BR1 vector encoding mCherry with and without the PFF sequence, immunohistochemical staining of mCherry and TfR (highly expressed in BECs) was performed, focusing on the cerebral cortex and hippocampus (Fig. 4A). Co-localization of mCherry and TfR confirmed successful transduction of BECs. However, compared to previous observations [13], the transduction efficiency in BECs was lower than anticipated. Furthermore, the stained brain slices exhibited nonspecific, scattered dots of mCherry staining, regardless of whether mice were injected with AAV-BR1 or PBS (Fig. 4A, indicated by white arrows). The unspecific staining was often seen as small circular dots and interpreted as lipofuscin granules with a diameter in the range of a few microns, which were distinguishable from the solid cytosolic labeling of transduced cells. Therefore, the low specificity of the staining did not allow for the assessment of protein secretion. Notably, initial staining was performed without tyramide amplification, yielding similar observations. However, all staining was repeated using the tyramide amplification to ensure that the detection of secreted mCherry was not overlooked. Despite the C-*Ocln* previously being described as endothelial specific, we observed several mCherry-positive cells having neuronal morphology, especially in the CA3 region of the hippocampus (Fig. 4B). This was confirmed when double-labeling the cells with mCherry and NeuN (neuronal marker). mCherry-positive neurons could also be detected in the cerebral cortex, indicating that the C-*Ocln* promoter was not as endothelial-specific as anticipated (Fig. 4B). The images did not reveal any additional signs of mCherry adjacent to cells transduced with AAV-BR1-C-*Ocln-*PFFmCA compared to mice transduced with AAV-BR1-C-*Ocln*-mCA, providing no clear evidence of secretion or unspecific uptake by surrounding cells. To further investigate luminal and abluminal secretion, mCherry protein concentration was measured by ELISA in plasma and CSF, respectively. Despite the high sensitivity of the assay, mCherry could not be detected in the low volume of CSF extractable from mice, nor in the plasma (data not shown). mCherry is expected to be localized in vesicles when entering the secretion pathway; however, this could not be observed in mCherry-positive mBECs or neurons in the cerebral cortex at higher resolution (Fig. 4C). Again, no differences were observed in cells transduced by either of the constructs.

### mCherry secretion is possibly enhanced by the inclusion of the PFF sequence

**Fig. 5:**
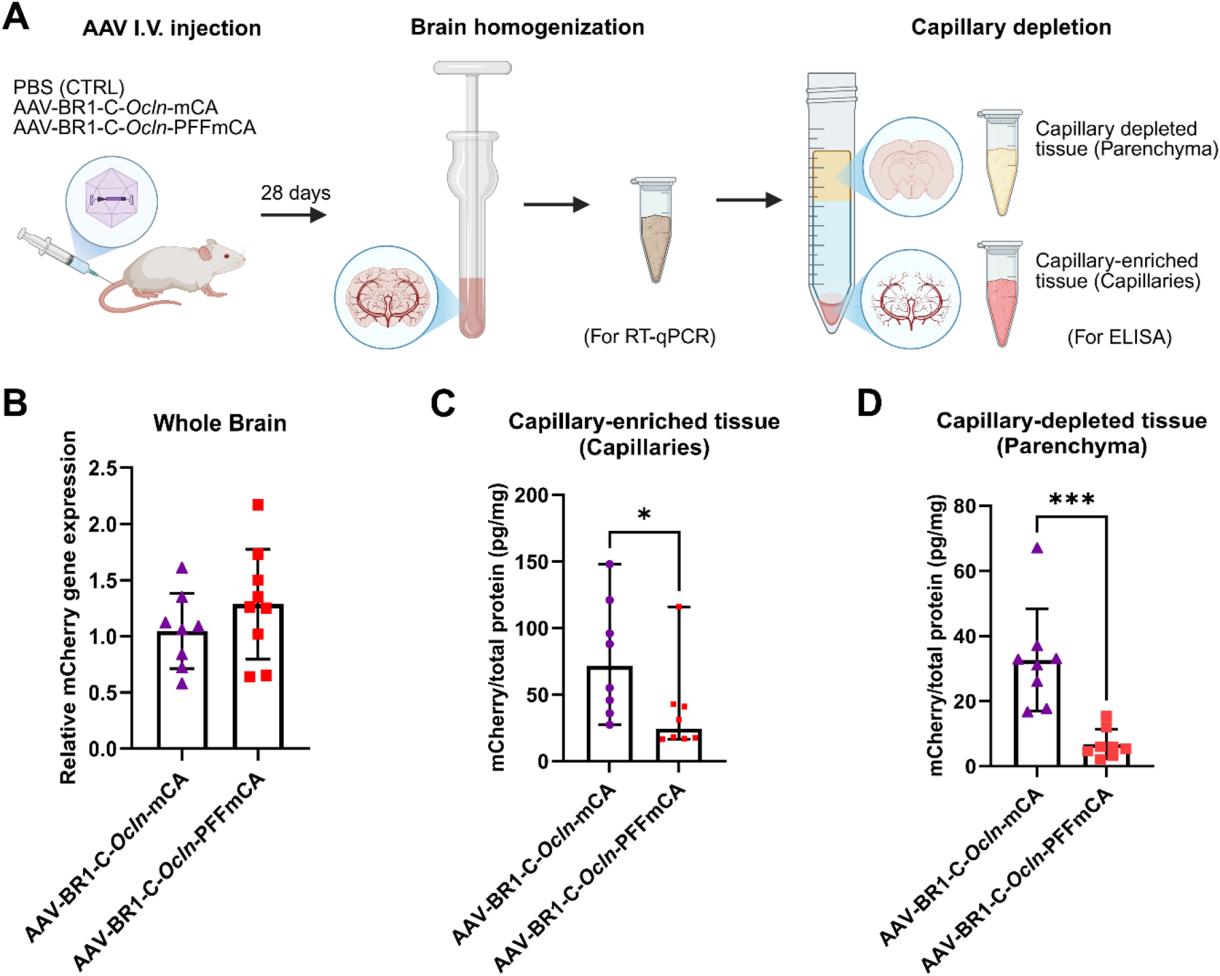
Identification of mCherry protein quantities in capillary-depleted brain fractions after viral transduction in mice. **(A)** A simplified schematic of the experiment. Purified AAVs or PBS were administered IV through the tail vein. Whole brains were harvested 28 days later and homogenized for subsequent RT-qPCR analysis, or capillary-depleted to separate the capillary-enriched tissue (capillaries) from the capillary-depleted tissue (parenchyma). The mCherry protein content of the two fractions was determined using ELISA. Created with BioRender.com. **(B)** The relative gene expression of mCherry in brain homogenate shows no significant difference between mice transduced with AAV-BR1-C-*Ocln*-mCA (*n* = 8) or AAV-BR1-C-*Ocln*-PFFmCA (*n* = 8). No mCherry mRNA was detected in CTRL mice (*n* = 5). Data are presented as mean ± standard deviation. Student’s t-test was used for statistical analysis (*P* = 0.265). **(C)** mCherry protein concentration in capillary-enriched tissue (capillaries) from mice transduced with AAV-BR1-C-*Ocln*-mCA (*n* = 8) is significantly higher than mCherry protein concentrations in capillaries from mice transduced with AAV-BR1-C-*Ocln*-PFFmCA (*n* = 8), suggesting secretion of mCherry due to the PFF fragment. No mCherry protein was detected in CTRL mice (*n* = 2). Data are presented as the median with 95% CI, and raw ELISA absorbances are shown in Fig. S3. A Mann-Whitney U test was used for statistical analysis (*P < 0.05). **(D)** The mCherry protein concentration in capillary-depleted tissue (parenchyma) from mice transduced with AAV-BR1-C-*Ocln*-PFFmCA is significantly lower than in the parenchyma of mice transduced with AAV-BR1-C-*Ocln*-mCA, likely suggesting secretion and subsequent clearance of mCherry from the brain parenchyma. Data are presented as mean ± standard deviation (*n* = 8). A Student t-test was used for statistical analysis (***P < 0.001). In B-D, values for the CTRL are not included, as no mCherry was detectable by either RT-qPCR or ELISA

We anticipated that the PFF sequence would result in higher quantities of mCherry in the brain parenchyma, due to abluminal secretion, compared to mice transduced with the construct that did not contain the PFF sequence. Therefore, we compared the mRNA expression of mCherry in whole brain homogenates of mice transduced with AAV-BR1-C-*Ocln-*PFFmCA to mice transduced with AAV-BR1-C-*Ocln*-mCA. Likewise, the mCherry protein concentrations in brain capillary-enriched tissue (capillary) vs. brain capillary-depleted tissue (parenchyma) were measured (Fig. 5A). No mCherry mRNA (data now shown) or protein was detected in CTRL mice (Fig. S3). As expected, the relative expression of mCherry in whole brain homogenate was comparable between the two groups of mice injected with either AAV-BR1-C-*Ocln*-mCA or AAV-BR1-C-*Ocln-*PFFmCA (Fig. 5B), indicating equal viral transduction efficiency and equal gene expression levels between the two vectors.

Capillary tissue from mice transduced with AAV-BR1-C-*Ocln*-mCA and AAV-BR1-C-*Ocln*-PFFmCA had median and 95% CI of mCherry protein quantities of 77.1 [41.0, 113.3], and 37.5 [9.5, 65.6] pg/mg total protein, respectively (Fig. 5C), showing a significant reduction in mCherry protein concentrations in capillaries of mice transfected with AAV-BR1-C-*Ocln*-PFFmCA, indicating secretion of mCherry from mBECs when transduced with the vector construct containing the PFF sequence. mCherry protein concentrations in the brain parenchyma were likewise significantly lower when mice were transduced with AAV-BR1-C-*Ocln-*PFFmCA (6.8 ± 4.5 pg/mg total protein) compared to mice transduced with AAV-BR1-C-*Ocln*-mCA (32.6 ± 15.7 pg/mg total protein) (Fig. 5D). This observation was, however, unexpected since the mCherry proteins were expected to be abluminal secreted thereby increasing the protein concentration in the brain parenchyma. The off-target transduction of neurons observed in the histochemical staining (Fig. 4B) could, however, explain why the mCherry protein quantities are higher in the brain parenchyma when mice were injected with the AAV-BR1-C-*Ocln*-mCA vector. Furthermore, the lower protein quantity of mCherry in the brain parenchyma of mice injected with AAV-BR1-C-*Ocln-*PFFmCA strongly indicates degradation and/or clearance of secreted mCherry from the brain, which highlights the challenge of detecting the secretion of recombinant proteins after in vivo transduction.

### The PFF sequence does not promote differential microglia activation near mCherry-positive cells

**Fig. 6:**
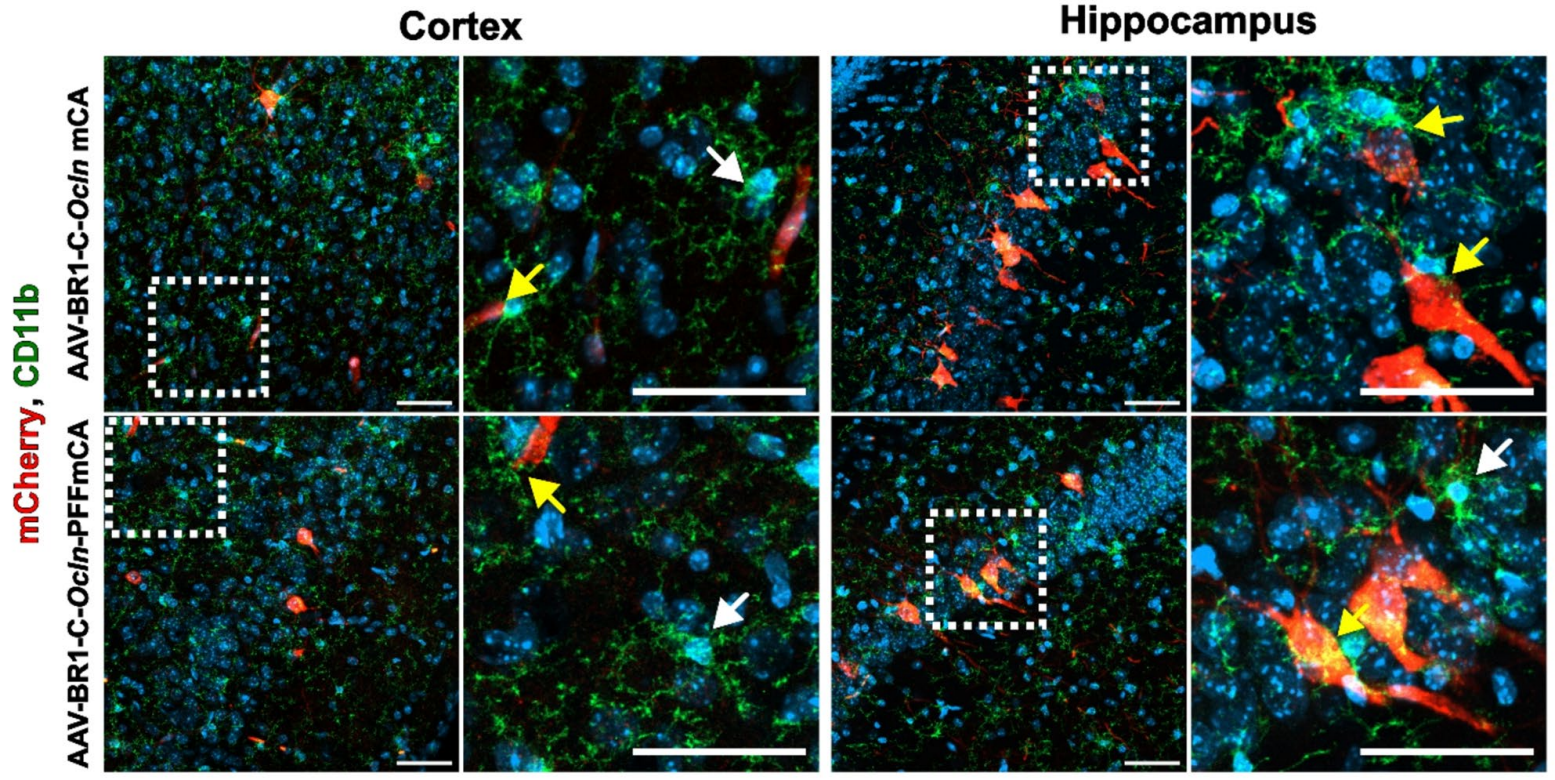
Microglia interact with transduced cells independently of the vector constructs. Microglial cells were identified using the microglial marker cluster of differentiation 11b (CD11b) (green) to study their interaction with mCherry-positive cells (red) in the cerebral cortex (left) and hippocampus (right). Framed areas marked with dashed lines show microglia (white arrows), and the interaction between microglia and transduced cells (yellow arrow) in higher magnification. No distinct differences are observed between mice transduced with AAV-BR1-C-*Ocln*-mCA and AAV-BR1-C-*Ocln*-PFFmCA. The nuclei are counterstained with DAPI (blue). Scale bar = 50 μm. Images represent mice transduced with either AAV-BR1-C-*Ocln*-mCA (*n* = 3) or AAV-BR1-C-*Ocln*-PFFmCA (*n* = 3)

Although we were unsuccessful in measuring or visually observing the abluminal secretion of mCherry, we wondered whether the extracellular presence of a non-endogenous recombinant protein, such as mCherry, could have triggered the activation of microglial cells adjacent to the transduced cells. Therefore, double staining for mCherry and CD11b (marker of resting and activated microglia and monocytes/macrophages) was performed in both the cortex cerebri and the hippocampus (Fig. 6). No apparent differences in microglia reactivity and abundance were observed when comparing mice transduced with vectors with and without the PFF sequence. Microglia interacted with mCherry-positive cells in both conditions, suggesting that the presence of extracellular mCherry did not lead to increased activation.

### The PFF sequence results in higher abluminal secretion of mCherry, in vitro

**Fig. 7:**
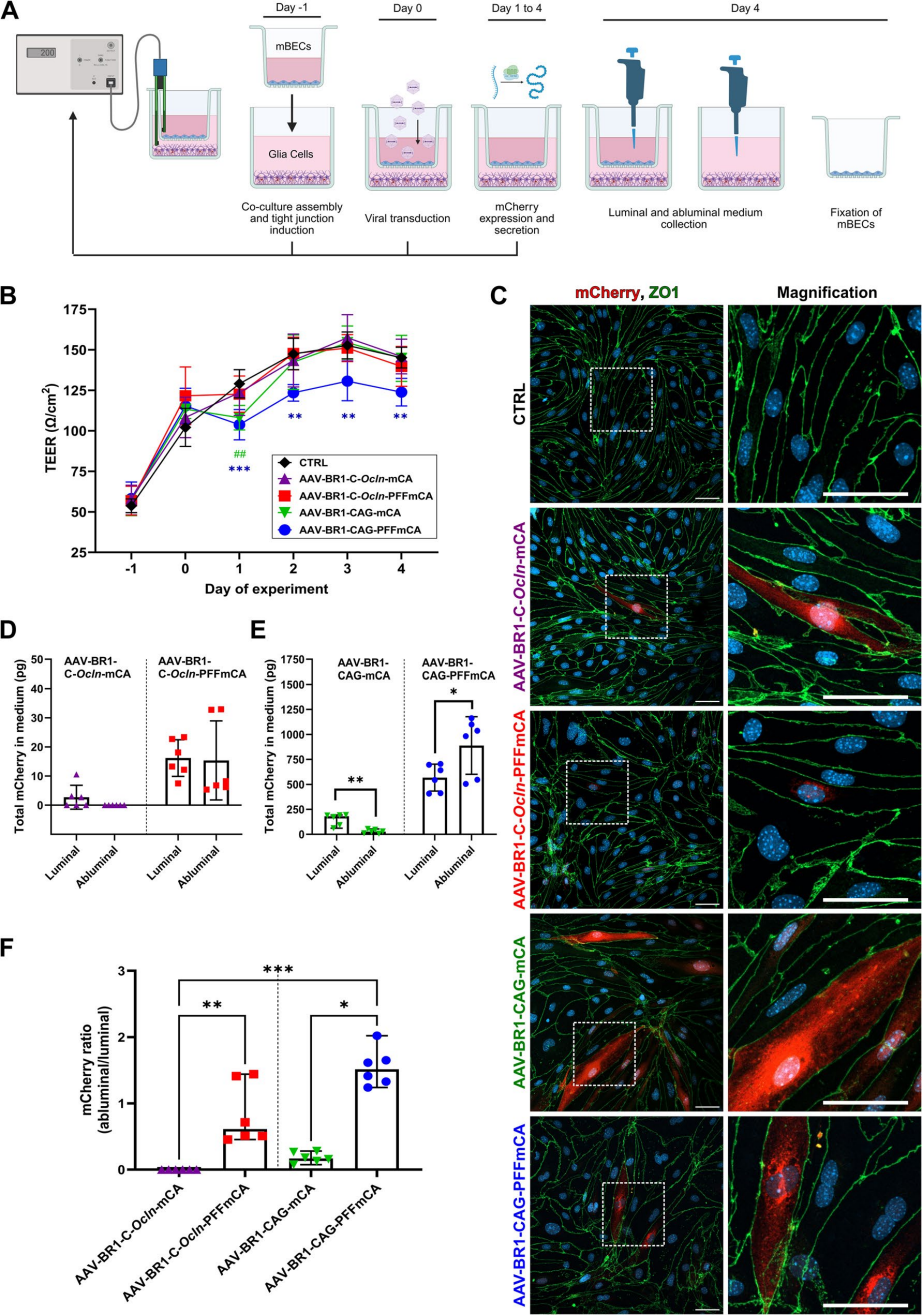
Viral transduction of mouse brain endothelial cells (mBECs) co-cultured with mixed glial cells. **(A)** Schematic of experimental design. Day − 1: Co-cultures of mBECs and mixed glia cells were established and stimulated to induce tight junctions. Day 0: mBECs were transduced by the addition of the viral vector constructs. Non-transfected mBECs are referred to as controls (CTRL). Day 4: The medium was harvested for ELISA, and mBECs were fixed for immunocytochemistry. Trans-endothelial electrical resistance (TEER) measurements were conducted from day − 1 through day 4. Created with BioRender.com. **(B)** TEER is lower in mBECs transduced with either AAV-BR1-CAG-mCA(^##^) or AAV-BR1-CAG-PFFmCA(***) on day 1 compared to CTRL. From day 2, mBECs transduced with AAV-BR1-CAG-mCA return to TEER values comparable to CTRL, while mBECs transduced with AAV-BR1-CAG-PFFmCA remain significantly decreased (**). Data are depicted as mean ± SD (*n* = 6). Statistical analysis was conducted using a mixed-effects model (REML) with the Dunnett multiple comparison test. **(C)** Transduced mBECs were identified using immunocytochemical staining against mCherry (red) and the tight junction marker ZO-1 (green). The nuclei are counterstained with DAPI (blue). Scale bar = 40 μm. Representative images (*n* = 3). **(D + E)** mCherry concentrations measured in luminal and abluminal medium collected from transduced mBECs. mCherry is detected in the luminal medium from mBECs transduced with both constructs not containing the PFF sequence, suggesting nonspecific release of mCherry. However, including the PFF sequence in the vector construct increases abluminal secretion of mCherry, especially when combined with the CAG promoter. No mCherry was detected in CTRL (*n* = 5). All data are presented with a median and 95% CI (*n* = 6), except AAV-BR1-CAG-PFFmCA, which is presented with mean ± SD (*n* = 6). Raw ELISA absorbances are shown in Fig. S3. Statistical analysis was conducted using the Mann-Whitney U test or Student’s T-test, as appropriate. **(F)** The ratio of secreted mCherry between the luminal and abluminal chambers in each culture well was calculated from the data presented in D and E. mBECs transfected with AAV-BR1-CAG-PFFmCA and AAV-BR1-C-*Ocln*-PFFmCA have significantly higher ratios of abluminal secretion. Data are presented with median and 95% CI (*n* = 6). Statistical analysis was conducted using a Kruskal-Wallis test with Dunn’s multiple comparison post hoc test. Significance levels were *P < 0.05, **P < 0.01, ***P < 0.001

The transduction pattern using the endothelial-specific C-*Ocln* promoter differed from previous studies, which had shown overwhelming transduction of the vasculature [13]. Instead, we observed off-target transduction of neurons, which obscured the detection of potential abluminal secretion of mCherry by mBECs (Fig. 1). The vectors were therefore further investigated using an in vitro BBB model based on primary mBECs and mixed glial cells, where no neurons were present. Assuming some of the observed discrepancy in vivo between mRNA and protein quantity can be attributed to secretion, it remains unanswered whether the transduction of mBECs with a vector encoding the PFF sequence contributes to increased abluminal secretion, or if the secretion of mCherry by mBECs is mainly luminal, as previously reported using similar setups without the PFF sequence [4, 8]. Since the C-*Ocln* was less potent and endothelial-specific than expected, and because comparable studies have been performed using strong, ubiquitous promoters such as CMV and CAG [4, 8], we also incorporated the CAG promoter-driven mCherry constructs into the AAV-BR1 vector. Primary mBECs were therefore transduced with the AAV-BR1 vector containing either the C-*Ocln*-mCA, C-*Ocln-*PFFmCA, CAG-mCA, or CAG-PFFmCA vector constructs. These were compared to non-transduced mBECs (CTRL). Four days post-transduction, the polarized secretion of mCherry was investigated by measuring mCherry protein concentrations in the cell culture medium collected from the upper and lower chambers in the in vitro BBB model, representing the luminal (blood) and abluminal (parenchyma) sides, respectively (Fig. 7A). mBECs were fixed to examine the intracellular sorting of mCherry, and during the experiment, the effect of transduction on the mBECs barrier integrity was measured using TEER (Fig. 7A). In general, the barrier integrity of the mBECs was not affected by the addition of AAV-BR1, however, mBECs transduced with vectors containing the CAG promoter had an initial decrease in barrier integrity on day 1, which persisted in cultures transduced with AAV-BR1-CAG-PFFmCA throughout the experiment but were quickly normalized for cultures transduced with AAV-BR1-CAG*-*mCA (Fig. 7B). Using immunocytochemical staining, no mCherry was detected in CTRL cells, but mCherry was observed evenly distributed through the cytosol of mBECs transduced with the vector constructs, not including the PFF sequence (AAV-BR1-CAG-mCA and AAV-BR1-C-*Ocln*-mCA), corresponding well with an intracellular distribution of mCherry (Fig. 7C). When the PFF sequence was included in the vector constructs (AAV-BR1-CAG-PFFmCA and AAV-BR1-C-*Ocln*-PFFmCA), mCherry was predominantly localized around the nucleus, indicating entry into the secretory pathway and packaging in vesicles (Fig. 7C). More transduced cells were observed when the mBECs were transduced with vector constructs controlled by the CAG promoter.

No mCherry protein was detected in the CTRL cultures (Fig. S3), while mBECs transduced with AAV-BR1-C*-Ocln*-mCA only had low and almost non-detectable luminal secretion in 50% of the cultures (1.3 pg 95% CI [-1.6, 7.0]). In contrast, no abluminal secretion was observed (Fig. 7D). mBECs transduced with AAV-BR1-C-*Ocln*-PFFmCA revealed that the combination of the PFF sequence and C-*Ocln* promoter resulted in secretion of mCherry to both the luminal (15.7 pg 95% CI [9.6, 22.7]), and abluminal chamber (7.5 pg 95% CI [1.2, 29.6]) (Fig. 7D). Including the PFF sequence therefore provides a more favorable ratio of abluminal secretion than that reported previously [4, 8]. mBECs transduced with the AAV-BR1-CAG-mCA resulted in higher mCherry concentrations in both the luminal (181.0 pg 95% CI [88.7, 210.5]) and abluminal (27.2 pg 95% CI [9.1, 48.5]) chambers than observed with the C-*Ocln* promotor, with the concentration of mCherry in the luminal chamber being significantly higher than that observed in the abluminal chamber (Fig. 7E). This was surprising as this vector does not contain the PFF sequence but could suggest that high quantities of intracellular mCherry are nonspecifically released mainly in the luminal chamber, or that the cell undergo lysis because of the transduction process, as also observed in the initial transfection’s studies with HEK 293T and bEnd.3 cells. Transduction with the AAV-BR1-CAG-PFFmCA construct leads to statistically higher mCherry quantities in the abluminal chamber (889.1 ± 288.1 pg) compared to the luminal chamber (567.6 ± 135.1 pg) (*P < 0.05) (Fig. 7E). Furthermore, when the ratio between luminal and abluminal secreted mCherry within each cell culture well is compared between the groups, the medium from cells transduced with AAV-BR1-CAG-PFFmCA has the highest ratio of abluminal secretion (1.51, 95% CI [1.24, 2.02]), which is statistically significant from mBECs transfected with AAV-BR1-CAG-mCA (0.17, 95% CI [0.08, 0.28]) (*P < 0.05) (Fig. 7F). Likewise, a significant difference was seen when mBECs were transfected with AAV-BR1-C-*Ocln*-PFFmCA (0.61, 95% CI [0.46, 1.44]) compared to AAV-BR1-C-*Ocln*-mCA (0, 95% CI [0.00, 0.00]) (**P < 0.01), suggesting that the PFF sequence allows for abluminal secretion independent of the promotor choice (Fig. 7F). A combination of the CAG promoter and the PFF sequence resulted in the highest ratio of abluminal secretion, and the quantity of abluminal mCherry was 100 times higher than the observed abluminal secretion from mBECs transduced with vectors encoding the C-*Ocln* promoter.

## Discussion

By specifically developing and describing strategies for abluminal protein secretion, our study aimed to expand upon prior work using AAV-BR1-directed gene therapy [5, 6], providing proof-of-concept measurements of abluminal secretion by BECs. The correct processing of the vector constructs was confirmed in vitro, and approximately one-third of the secreted mCherry protein was processed by the furin protease in bEnd.3. This may possibly lower the applicability of BEC-directed gene therapy with peptides that require post-translational targeted cleavage for functionality. BEC-specific transduction was attempted in vivo by including the endothelial-specific C-*Ocln* promoter in the vector construct. We aimed to specifically investigate the abluminal secretion of mCherry by BECs, which involved a minimum contribution of mCherry production by off-target cells located in the parenchyma. Off-target transduction of neurons is commonly seen when using the CAG promoter [5, 6, 13]. To our surprise, the C-*Ocln* promoter was not as endothelial-specific as anticipated, resulting in several off-target transduced neurons, together with low transduction efficiency, which hindered a solid verification of abluminal secreted mCherry. However, the analysis of mice transduced with AAV-BR1 indicated that including a PFF sequence into the vector construct likely led to the secretion of mCherry from both BECs and neurons. Additional in vitro studies on the potential of the PFF sequence revealed increased abluminal secretion of mCherry from primary mBECs when the PFF sequence was incorporated into the vector constructs, particularly in combination with the CAG promoter. Studying the specific secretion of a recombinant protein following *vivo* transduction of only BECs remains a challenge due to off-target transduction of neurons, despite the use of an endothelial-specific promoter in the vector construct. This is particularly problematic, as previous observations [5, 6, 13] suggest that the CAG promoter would likely exacerbate this issue.

### Vector design is essential for high mCherry expression and abluminal-directed secretion

The CAG promoter provided higher secretion of mCherry from immortalized cell lines relative to the *C-Ocln* promoter. When investigating polarized secretion in primary cell co-cultures of mBECs and mixed glia cells transduced with AAV-BR1, similar observations were made. Medium from mBECs transduced with AAV-BR1-CAG-PFFmCA had 30 times higher luminal and 100 times higher abluminal concentrations of mCherry than medium from mBECs transduced with the C-*Ocln* promoter. Applications requiring high expression can therefore benefit from using the CAG promoter. Importantly, incorporating the PFF sequence together with the C-*Ocln* promoter likewise resulted in a higher abluminal secretion of mCherry in primary mBECs compared to that previously reported in vitro [4, 8]. Likewise, the transduction of mBECs in vitro with AAV-BR1 encoding the CAG promoter coupled with only the PDGF-B fragment of the PFF sequence fused to IL-10 has also shown significantly higher abluminal secretion in our lab in another experiment (Laczek et al. in preparation), indicating the PDGF-B fragment can direct different proteins of interest towards the abluminal chamber. Even though preferential abluminal secretion of a transgene following gene therapy has been described in immortalized cell lines of mBECs [21], this is the first time that a similar tendency is described in a culture of primary cells, indicating that the inclusion of a PFF sequence can potentially enhance secretion at the abluminal surface of BECs. Future studies could expand on the use of different signal sequences or the impact of fusion proteins to enhance the abluminal secretion pathway in mBECs, as has been done in various biotherapeutic production schemes, such as monoclonal antibody production [22, 23]. This could enable the identification of optimal efficiency in targeted gene therapy of mBECs as therapeutic protein factories with high abluminal secretion. The fusion protein sequence consisted of the signal sequence from PDGF-B and its following 10 amino acids. The rationale behind this approach was to promote the correct recognition of the signal sequence by the signal recognition particle, the insertion of the protein sequence into the correct polarized secretion pathway, and, finally, correct cleavage by the signal peptidase. However, all the above mechanisms and their influences on obtaining specific abluminal secretion are unknown, so understanding each of them would require a comprehensive study to be fully described, especially since the polarized secretion of endothelial cells is poorly understood [10–12]. It is, therefore, possible that the preferential abluminal secretion observed in mBECs transduced with AAV-BR1-CAG-PFFmCA, could be achievable by only using the signal sequence of PDGF-B, without including additional amino acids, and that the cleavage of the signal sequence would be performed in the correct location when fused to a sequence of an N-terminally sensitive protein, thereby not requiring a furin cleavage site. As approximately 70% of the secreted mChery proteins from bEnd.3 were not cleaved by furin, the investigations, as mentioned earlier, could provide valuable information, including the PFF sequence together with an N-terminally sensitive peptide, which might not be an optimal strategy, as this could prevent a significant proportion of the proteins from functioning. Conversely, another approach to investigate abluminal secretion could involve adding a more extended PDGF-B sequence following the signal peptide, potentially further increasing abluminal secretion. This strategy, however, also carries the risk of disrupting the function of the fused protein entirely due to the interaction of the PDGF-B fragment with its receptor, which is expressed by pericytes [24].

### BBB integrity following AAV-BR1 gene therapy

While investigating polarized secretion in vitro in primary co-cultures of mBECs and glial cells, a decrease in barrier integrity was observed in cells transduced with AAV-BR1-CAG-PFFmCA. It is essential to highlight that TEER values never descended below levels that would compromise BBB integrity, facilitating the high passage of even small molecules [16, 17], indicating that the diffusion of mCherry from the luminal chamber through the barrier is unlikely to be the cause of higher abluminal mCherry concentrations. The AAV-BR1 vector is known to undergo passage through the mBECs cell layer; however, a previous study has shown this phenomenon does not lead to a significant increase in mRNA expression in glial cells of the recombinant protein encoded by the vector [5], suggesting that possible transduction of glial cells in the abluminal chamber could not explain the high concentrations of mCherry. Abluminal secretion by mBECs, therefore, seems to be the main reason for the high concentrations of mCherry in the abluminal chamber. The underlying mechanisms contributing to reduced barrier integrity, however, remain elusive. Considering that the decreased TEER values were only observed when mBECs were transduced with AAV-BR1-CAG-PFFmCA, and not previously observed in other experiments using the same promoter and a native signal for secretion [5, 6], a potential reason for this could be increased abluminal secretion or heightened furin protease activation, which might disrupt the natural cellular processes of BECs.

### Tracking of mCherry secretion in vivo

The strength of the C-*Ocln* promoter was expected in vivo, where a reduction in off-target transduction of neurons and lung tissue was anticipated, based on a previous report [13]. However, neuronal expression of mCherry was nonetheless present using the C-*Ocln* promoter, which is why protein measurements of mCherry in capillary-depleted brain tissue (parenchyma) were significantly higher when mice were transduced with the vector without the PPF sequence. Off-target transduced neurons secreting mCherry post-transduction likely resulted in an increased content of mCherry in brain parenchymal tissue, despite similar mRNA expression levels of mCherry being measured in whole brain homogenate. It is, however, essential to note that equal levels of mRNA do not necessarily translate into equivalent protein generation, particularly when one of the proteins is destined to undergo processing through a secretory pathway, but a strong correlation often exists between two measures of the same gene [25]. It is possible that a significant portion of the observed difference in intracellular mCherry content within both capillary-enriched tissue (capillaries) and capillary-depleted tissue (parenchyma) can be attributed to secretion. The use of Proximity Extension Assay-based Olink technologies for direct mouse CSF measurements [26] or optimized HPLC-MS methods could circumvent issues related to low volume and CSF protein content [27].

### Off-target in vivo expression

Pyramidal neurons express occludin [28], which could explain their high off-target expression. Pyramidal neurons are located in the hippocampus, so the high transduction observed in this area could result from the C*-Ocln* promoter not alleviating off-target specificity specifically in these neurons. Another possibility is that the targeted specificity associated with promoter types is affected by regulatory elements of gene expression, which function differently when incorporated into episomal DNA rather than chromatin [29]. The results of this study indicated that both transduced neurons and mBECs were capable of secreting post-transduction, as evidenced by comparing mRNA expression with measured mCherry protein content. The in vivo results using the AAV-BR1 and C-*Ocln* promoter need to be considered in the context of the varying results of cellular AAV-BR1 tropism between laboratories [5–7, 13]. The target receptor of AAV-BR1 remains unknown; however, tropism between mouse strains for LY6A, a receptor for AAV-PHP.B, substantially impacts brain transduction of other brain-targeted AAV variants [30]. Although C57BL/6 mice were also used in the study investigating the C-*Ocln* promoter [13], other factors, such as the commercial breeding facility, laboratory environment, age at transduction, or sex of the mice, may have affected the expression of the AAV-BR1 receptor, leading to the observed discrepancies between studies.

### BECs as protein factories

Genetically modifying the interface between the blood and the brain to enable secretion of otherwise BBB-impermeable proteins is an attractive drug delivery strategy. The strategy, however, involves the specific transduction of the brain, rather than peripheral organs, which can be achieved using the AAV-BR1 viral vector [5, 6]. The drug delivery strategy was recently tested in a mouse model of Niemann-Pick disease and found to alleviate the neurological symptoms and lipid deposition in the AAV-BR1-treated mice [6]. Genetically modified BECs might, however, secrete the recombinant protein in both luminal and abluminal directions, which in some cases might be a problem, as some neurotropic factors, such as growth hormone 1, have been shown to cause pancreatic cancer in monkeys when present in the circulation [31]. Including a signal sequence that directs the recombinant protein towards an abluminal secretion pathway is therefore highly relevant. To specifically study the secretion pathway of BECs in vivo, we needed to develop a vector strategy targeting only these cells. Unfortunately, we were unable to replicate the specificity of the BECs for the C-*Ocln* promoter, as reported previously [13]. However, in vitro results strongly suggest that including the signal sequence of PDGF-B in the vector design promotes abluminal secretion. When aiming solely to achieve high concentrations of the recombinant protein within the brain parenchyma, off-target transduction of neurons can, however, be advantageous as it increases the concentration of the recombinant protein inside the brain parenchyma. Therefore, future therapeutic applications for treating brain diseases would benefit from incorporating a vector strategy that combines the PDGF-B signal sequence with the CAG promoter, thereby achieving strong transduction efficiency and increased abluminal secretion from BECs.

## Conclusions

This study aimed to develop a vector strategy to increase the abluminal protein secretion of mCherry in vivo by transducing BECs using the brain-specific AAV-BR1 vector. BEC-specific transduction was attempted in vivo by including the endothelial-specific C-*Ocln* promoter in the vector construct to minimize off-target transduction of neurons. A PFF sequence was incorporated in the vector design to direct the recombinant protein towards an abluminal secretion pathway. Mice transduced with the AAV-BR1-C-*Ocln-*PFFmCA vector facilitated mCherry expression and possibly secretion when coupled with the PFF sequence. The C-*Ocln* promoter was, however, not found to be as endothelial-specific as anticipated, resulting in off-target transduced neurons that obscured the measurement of abluminal mCherry concentrations. Using an in vitro BBB model, the AAV-BR1 vector constructs containing the PFF sequence demonstrated enhanced abluminal secretion of mCherry, particularly when combined with the CAG promoter. Together, these observations show that including the PFF sequence in the vector construct targets the protein of interest for abluminal secretion, which is amplified when combined with the strong CAG promoter.

## Supplementary Information

Below is the link to the electronic supplementary material.

Supplementary Material 1

## Data Availability

All data generated and analyzed during this study are included in this published paper. All datasets are available from the corresponding author upon reasonable request.

## Abbreviations

BBB: Blood-brain barrier
BECs: Brain endothelial cells
CAG-Luc: CAG-Luciferase
CAG-mCA: CAG-mCherry-Avitag-WPRE
CAG-PFFmCA: CAG-PDGF-B-3XFLAG-Furin-mCherry-Avitag-WPRE
CD11b: Cluster of differentiation 11b
CNS: Central nervous system
CSF: Cerebrospinal fluid
C-*Ocln*: CMV enhancer-occludin
C-*Ocln*-mCA: C-*Ocln*-mCherry-Avitag-WPRE
C-*Ocln*-PFFmCA: C-*Ocln*-PDGF-B-3XFLAG-Furin-mCherry-Avitag-WPRE
cDMEM: complete Dulbecco’s Modified Eagle Medium
CI: Confidence interval
GLP-1: Glucagon-like peptide 1
IV: Intravenous
mBECs: mouse brain endothelial cells
PBS: Phosphate-buffered saline (PBS)
PPBS: Potassium phosphate buffered saline
PDGF-B: Platelet-derived growth factor subunit B
PFF: PDGF-B-3XFLAG-Furin
RT: Room temperature
TEER: Trans-endothelial electrical resistance
TfR: Transferrin receptor
TBS-T: Tris-buffered saline with Tween® 20
DAPI: 4′,6‐diamino‐2‐phenylindole
