# Supplementary material for: A novel vector design targeting the blood-brain barrier with the brain-specific AAV-BR1 vector enables abluminal protein secretion from brain endothelial cells in vitro
Source: Fluids Barriers CNS. 2025 Dec 12;23:7. doi: 10.1186/s12987-025-00738-6 (PMC12817821; doi:10.1186/s12987-025-00738-6)
Supplement: Supplementary file 1 — Supplementary Material 1 [file 12987_2025_738_MOESM1_ESM.pdf]

SUPPLEMENTARY FILE

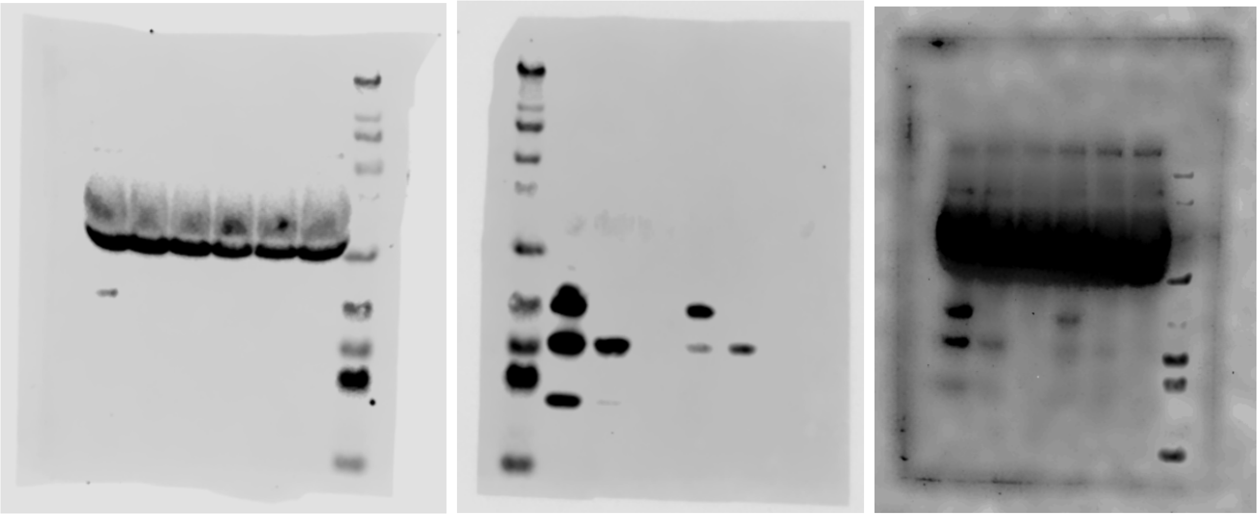

Figure 1S. Uncropped Western blots (WB) correspond to those displayed in Fig. 3A. Right side Anti-FLAG staining, middle and left side Anti-Avitag Staining of cell culture medium from transfected HEK 293T and bEND.3 cells. The cell culture medium contains 10% FCS, which is why the unspecific band seen especially on the right and left membranes, which have the highest exposure times, probably corresponds to serum proteins like serum albumin.

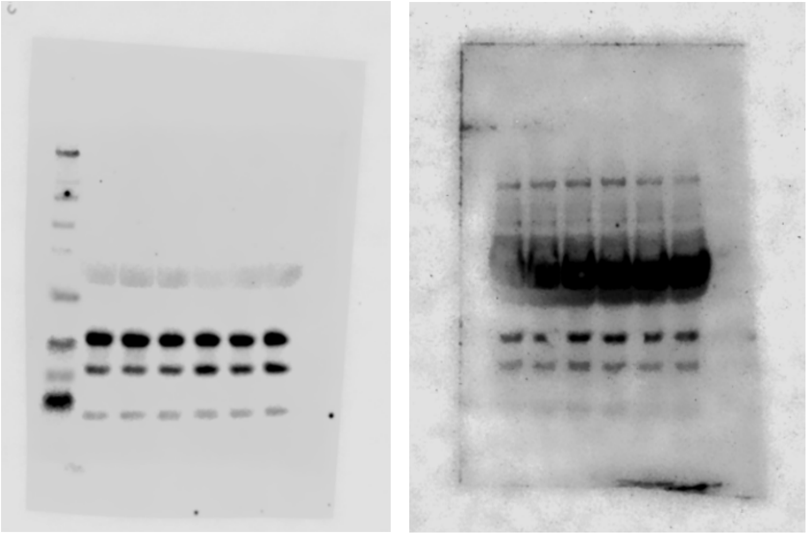

Figure S2, Uncropped Western blots (WB) corresponding to the data displayed in Fig. 3C and D. Left side cell culture medium from bEnd.3 cells transfected with the CAG-PFF-mCA vector construct (n=6) and right-side cell culture medium from bEnd.3 cells transfected with the C-*Ocln*-PFF-mCA vector construct Uncropped Western blots (WB) corresponding to those displayed in Fig. 3A. (n=6).

A

First ELISA on mouse brain tissue (Fig. 5)

Standard Curve Absorbance:		Concentration:
3,6658	3,5347	400
1,9575	1,8375	200
1,0887	1,0041	100
0,5797	0,5269	50
0,3186	0,2933	25
0,1765	0,1700	12,5
0,1135	0,1155	6,25
0,0568	0,0554	0

Capillary enriched tissue absorbance duplicates		
CTRL	0,1584	0,1513
CTRL	0,1345	0,1398
C-Ocln-mCA	0,4051	0,3987
C-Ocln-mCA	0,6083	0,6077
C-Ocln-mCA	0,7266	0,7124
C-Ocln-PFFmCA	0,3445	0,3559
C-Ocln-PFFmCA	0,3594	0,3153
C-Ocln-PFFmCA	0,4673	0,4881

Capillary depeleted tissue absorbance duplicates		
CTRL	0,1567	0,1367
CTRL	0,1360	0,1450
C-Ocln-mCA	0,9076	0,8814
C-Ocln-mCA	1,0514	1,0709
C-Ocln-mCA	1,6021	1,6565
C-Ocln-PFFmCA	0,3257	0,3005
C-Ocln-PFFmCA	0,3372	0,3091
C-Ocln-PFFmCA	0,3658	0,3765

Tissue-buffer (N-PER) absorbance (values should be substracted from absorbance measuements of samples)	
0,1276	0,0731

Second ELISA on mouse brain tissue (Fig. 5)

Standard Curve Absorbance:		Concentration:
2,9241	2,6524	400
1,3881	1,4002	200
0,7089	0,7369	100
0,3857	0,4264	50
0,2260	0,2571	25
0,1430	0,1589	12,5
0,1064	0,1316	6,25
0,0783	0,1162	0

Capillary depeleted tissue absorbance duplicates		
C-Ocln-mCA	0,8645	1,4474
C-Ocln-mCA	0,9247	1,4688
C-Ocln-mCA	1,0451	1,2024
C-Ocln-PFFmCA	0,5279	0,5894
C-Ocln-PFFmCA	1,2329	0,8329
C-Ocln-PFFmCA	0,9400	0,5022

Capillary depeleted tissue absorbance duplicates		
C-Ocln-mCA	1,1838	1,3072
C-Ocln-mCA	1,4903	1,7462
C-Ocln-mCA	0,8343	1,0621
C-Ocln-PFFmCA	0,4377	0,4811
C-Ocln-PFFmCA	0,7686	0,7741
C-Ocln-PFFmCA	0,5417	0,4439

Tissue-buffer absorbance (value should be substracted from absorbance measuements of samples)	
0,3187	0,3015

B

Third ELISA on mouse brain tissue (Fig. 5)

Standard Curve Absorbance:		Concentration:
3,4915	3,0898	400
1,8693	1,6031	200
0,9477	0,8734	100
0,5727	0,5189	50
0,2811	0,3115	25
0,2429	0,2211	12,5
0,2155	0,1541	6,25
0,1102	0,1145	0

Capillary enriched tissue absorbance duplicates		
C-Ocln-mCA	0,4321	0,4524
C-Ocln-mCA	0,5985	0,6027
C-Ocln-PFFmCA	0,3552	0,3428
C-Ocln-PFFmCA	0,4111	0,3947

Capillary depeleted tissue absorbance duplicates		
C-Ocln-mCA	0,9302	0,8977
C-Ocln-mCA	0,802	0,6542
C-Ocln-PFFmCA	0,4435	0,4404
C-Ocln-PFFmCA	0,3567	0,3375

Tissue-buffer (N-PER) absorbance (value should be substracted from absorbance measuements of samples)	
0,1602	0,1632

ELISA (1) performed on culture medium (Fig. 7)

Standard Curve Absorbance:		Concentration:
3,2153	2,9308	400
1,7985	1,6379	200
0,9623	0,8737	100
0,4855	0,4664	50
0,3062	0,2951	25
0,2476	0,3742	12,5
0,1846	0,1860	6,25
0,1368	0,1784	0

Samples of luminal medium duplicates		
C-Ocln-PFFmCA	0,4300	0,3976
C-Ocln-PFFmCA	0,4066	0,4340
CAG-mCA	2,0504	2,0023
CAG-mCA	2,1549	2,3006

Samples of abluminal medium duplicates		
C-Ocln-PFFmCA	0,3107	0,3153
C-Ocln-PFFmCA	0,3220	0,3057
CAG-mCA	0,3892	0,3814
CAG-mCA	0,4267	0,4281

Absorbance of medium (value should be substracted from absorbance measuements of samples)		
Luminal medium	0,1776	0,1653
Abluminal medium	0,1406	0,1360

ELISA (2) performed on culture medium (Fig. 7)

Standard Curve Absorbance:		Concentration:
3,0238	3,0216	400
1,5204	1,5105	200
0,7883	0,7805	100
0,4352	0,4412	50
0,2454	0,2525	25
0,1564	0,1521	12,5
0,1141	0,113	6,25
0,0883	0,0702	0

Absorbance of medium (value should be substracted from absorbance measuements of samples)		
Luminal medium	0,1355	0,1479
Abluminal medium	0,1057	0,1436

Samples of luminal medium duplicates			
CTRL	0,1674	0,1523	
CTRL	0,1704	0,1650	
CTRL	0,1421	0,1492	
CTRL	0,1692	0,1718	
C-Ocln-mCA	0,1993	0,1933	
C-Ocln-mCA	0,2228	0,2493	
C-Ocln-PFFmCA	0,3186	0,3317	
C-Ocln-PFFmCA	0,3742	0,3925	
CAG-mCA	2,0535	2,0508	
CAG-mCA	2,1039	2,0426	
CAG-PFFmCA	0,8341	0,8383	10X dilution
CAG-PFFmCA	0,8829	0,8939	10X dilution

Samples of abluminal medium duplicates			
CTRL	0,1351	0,1429	
CTRL	0,1425	0,1337	
CTRL	0,1353	0,1353	
CTRL	0,1313	0,1153	
CTRL	0,1439	0,1103	
C-Ocln-mCA	0,1552	0,1430	
C-Ocln-mCA	0,1204	0,1260	
C-Ocln-PFFmCA	0,2244	0,2136	
C-Ocln-PFFmCA	0,2241	0,2340	
CAG-mCA	0,3460	0,3209	
CAG-mCA	0,3093	0,3119	
CAG-PFFmCA	0,6915	0,7040	10X dilution
CAG-PFFmCA	0,6505	0,6914	10X dilution

ELISA (3) performed on culture medium (Fig. 7)

*Same plate as third ELISA performed on mouse brain tissue, therefore same calibration curve

Standard Curve Absorbance:		Concentration:
3,4915	3,0898	400
1,8693	1,6031	200
0,9477	0,8734	100
0,5727	0,5189	50
0,2811	0,3115	25
0,2429	0,2211	12,5
0,2155	0,1541	6,25
0,1102	0,1145	0

Absorbance of medium (value should be substracted from absorbance measuements of samples)		
Luminal medium	0,1817	0,2208
Abluminal medium	0,1656	0,2078

Samples of luminal medium duplicates			
CTRL	0,2231	0,1990	
C-Ocln-mCA	0,2162	0,2129	
C-Ocln-mCA	0,3398	0,3131	
C-Ocln-mCA	0,2743	0,2126	
C-Ocln-mCA	0,2220	0,2001	
C-Ocln-PFFmCA	0,3012	0,2861	
C-Ocln-PFFmCA	0,3604	0,3499	
CAG-mCA	0,8465	0,8665	
CAG-mCA	1,1513	1,2260	
CAG-PFFmCA	1,1350	1,1595	10X dilution
CAG-PFFmCA	0,9386	0,9313	10X dilution
CAG-PFFmCA	0,7718	0,7348	10X dilution
CAG-PFFmCA	0,7698	0,7500	10X dilution

Samples of abluminal medium duplicates			
C-Ocln-mCA	0,1253	0,1643	
C-Ocln-mCA	0,1557	0,1708	
C-Ocln-mCA	0,1685	0,1587	
C-Ocln-mCA	0,1458	0,1543	
C-Ocln-PFFmCA	0,2307	0,226	
C-Ocln-PFFmCA	0,2278	0,2442	
CAG-mCA	0,2698	0,2465	
CAG-mCA	0,2412	0,2326	
CAG-PFFmCA	0,9689	0,9703	10X dilution
CAG-PFFmCA	0,9282	0,9282	10X dilution
CAG-PFFmCA	0,5297	0,5383	10X dilution
CAG-PFFmCA	0,5722	0,5521	10X dilution

Figure S3. Raw ELISA data, including calibration data from in vivo and in vitro experiments. A) Three rounds of ELISA were conducted on mouse brain tissue separated into capillary-enriched tissue and capillary-depleted tissue. Duplicate absorbance values are displayed for CTRL (n=2), C-Ocln-mCA (n=8), and C-Ocln-PFFmCA (n=8). Tissue buffer

(N-PER) absorbances should be subtracted from all tissue samples. This raw data corresponds to the analyzed data presented in Fig. 5C and D. **B)** Three rounds of ELISA were conducted on cell culture medium from the luminal and abluminal chambers. Duplicate absorbance values are shown from cell culture medium from cells transduced with C-Ocln-mCA (n=6), C-Ocln-PFFmCA (n=6), CAG-mCA, CAG-PFFmCA (n=6), and from non-transduced cells (CTRL) (n=5). Absorbance from pure medium samples should be subtracted from all cell culture samples. The raw data corresponding to the analyzed data are presented in Fig. 7D and E. *Third ELISA on culture medium was performed on the same plate as the third ELISA performed on mouse brain tissue; therefore, the same calibration curve is presented twice.
